# Endogenous oxygen generating multifunctional theranostic nanoplatform for enhanced photodynamic-photothermal therapy and multimodal imaging

**DOI:** 10.7150/thno.38565

**Published:** 2019-10-15

**Authors:** Kun Wu, Honghai Zhao, Zhiquan Sun, Bing Wang, Xueying Tang, Yeneng Dai, Meixing Li, Qingming Shen, Hui Zhang, Quli Fan, Wei Huang

**Affiliations:** 1 Key Laboratory for Organic Electronics and Information Displays & Jiangsu Key Laboratory for Biosensors, Institute of Advanced Materials (IAM), Jiangsu National Synergetic Innovation Center for Advanced Materials (SICAM), Nanjing University of Posts & Telecommunications, Nanjing 210023, China.; 2 Jiangsu Key Laboratory of New Power Batteries, College of Chemistry and Materials Science, Nanjing Normal University, Nanjing 210097, China; 3 Shaanxi Institute of Flexible Electronics (SIFE), Northwestern Polytechnical University, Xi'an 710072, China

**Keywords:** oxygen generation, tumor hypoxia, photothermal therapy, photodynamic therapy, multimodal imaging

## Abstract

Phototherapy, including photothermal therapy (PTT) and photodynamic therapy (PDT), has been considered as a noninvasive option for cancer therapy. However, insufficient penetration depth, tumor hypoxia, and a single treatment method severely limit the effectiveness of treatment.

**Methods:** In this study, a multifunctional theranostic nanoplatform has been fabricated based on Au/Ag-MnO_2_ hollow nanospheres (AAM HNSs). The Au/Ag alloy HNSs were first synthesized by galvanic replacement reaction and then the MnO_2_ nanoparticles were deposited on the Au/Ag alloy HNSs by the reaction between Ag and permanganate (KMnO_4_), finally obtained the AAM HNSs. Then, SH-PEG was modified on the surface of AAM HNSs by the interaction of sulfhydryl and Au/Ag alloy, which improved the dispersibility and biocompatibility of the HNS. Next, the PDT photosensitizer Ce6 was loaded into AAM HNSs, benefiting from the hollow interior of the structure, and the AAM-Ce6 HNSs were obtained.

**Results:** The AAM HNSs exhibit broad absorption at the near infrared (NIR) biological window and remarkable photothermal conversion ability in the NIR-II window. The MnO_2_ nanoparticles can catalyze endogenous H_2_O_2_ to generate O_2_ and enhance the therapeutic effect of PDT on tumor tissue. Simultaneously, MnO_2_ nanoparticles intelligently respond to the tumor microenvironment and degrade to release massive Mn^2+^ ions, which introduce magnetic resonance imaging (MRI) properties. When AAM-Ce6 HNSs are loaded with Ce6, the AAM-Ce6 HNSs can be used for triple-modal imaging (fluorescence/photoacoustic/magnetic resonance imaging, FL/PAI/MRI) guided combination tumor phototherapy (PTT/PDT).

**Conclusion:** This multifunctional nanoplatform shows synergistic therapeutic efficacy better than any single therapy by achieving multimodal imaging guided cancer combination phototherapy, which are promising for the diagnosis and treatment of cancer.

## Introduction

Photothermal therapy (PTT) is a noninvasive therapy technique that transforms light into heat energy by photothermal agents and triggers cell damage through hyperthermia 1. Owing to its unique advantages of being minimally invasive, controllable and highly efficient, PTT has emerged as a promising modality for cancer treatment 2. In addition, because of its irreplaceable advantages of remote manipulation and high transparency in the “biological window”, near infrared (NIR) light has been widely used in the field of PTT. Compared with NIR-I (750~950 nm), the NIR-II window (1000~1350 nm), particularly in 1000~1100 nm range, has received increasing attention due to its deep tissue penetration, low scattering and higher maximum permissible exposure (MPE) to lasers (1 W/cm^2^ at 1064 nm laser, 0.33 W/cm^2^ at 808 nm laser) 3. Therefore, PTT in the NIR-II window has received increasing attention, especially for the treatment of buried tumors in deep tissue 4. In recent years, various NIR-II window photothermal agents have been developed, such as copper sulfide-based hybrid materials 5-8, gold-based composite materials 9-13 and organic photothermal nanoagents 14-16. Despite the excellent therapeutic effect of PTT in the NIR-II window, there are still some limitations. For example, PTT may not eliminate tumor cells completely, especially those located outside the irradiated area of light. Hence, residual tumor cells may be left on the treatment margin and may lead to tumor recurrence and metastasis 17, 18.

To address this problem, combining PTT with other treatments, such as photodynamic therapy (PDT) 19, 20, chemotherapy 21 and radiotherapy 22, has been proposed as an effective strategy to overcome the defects of other treatments, producing a synergistic effect and achieving better overall therapeutic outcomes 23. Like PTT, PDT is a phototherapy modality and has attracted tremendous attention as a cancer treatment 24. It utilizes reactive oxygen species (ROS) generated from photosensitizers under laser irradiation to promote cell apoptosis or necrosis. In principle, PDT is even more effective than PTT, where the ROS can facilitate the destruction of cancer cells. Furthermore, unlike the PTT effect, PDT is not suppressed by lower surrounding temperatures of the tumor 25. However, PDT is severely limited by the intrinsic hypoxic tumor microenvironment (TME) caused by the aggressive proliferation of tumor cells and the lack of blood supply in the interior of the tumor. Moreover, the constant consumption of O_2_ during the PDT process further intensifies tumor hypoxia and greatly impairs PDT efficiency 26, 27. Fortunately, several strategies have been proposed to alleviate the hypoxic TME by promoting the oxygenation of tumors. The photothermal effect induced by NIR photothermal agents could be utilized to increase intratumoral blood flow and subsequently improve the oxygenation status of the TME 28. Perfluorocarbon and perfluorohexane, which can dissolve a large amount of oxygen, have also been used as an oxygen reservoir to enhance the PDT efficacy 29, 30. More recently, research has reported that tumor hypoxia could be relieved by catalase 31, platinum nanoparticles 32 or manganese oxide (MnO_2_) nanoparticles, which can trigger the decomposition of endogenous hydrogen peroxide (H_2_O_2_) inside the tumor to produce O_2_ *in situ* and promote the therapeutic effect of PDT 33-36. In particular, nanotheranostics based on MnO_2_ have been extensively explored for their TME-responsive oxygen producing performance. On the one hand, MnO_2_ nanoparticles can be used to generate massive oxygen in TME. On the other hand, they can decompose in TME and generate Mn^2+^ ions, which can be used to enhance T1-magnetic resonance (MR) imaging contrast for tumor-specific imaging 37-40. For the precise treatment of tumors, it is necessary to develop theranostic nanoplatform with diagnostic and therapeutic functions 41. In addition to MR imaging guidance, computed tomography imaging 42, fluorescence imaging 43, photoacoustic and photothermal imaging 44, 45 can be applied as diagnostic methods for tumors. Different imaging technologies have their own advantages and disadvantages. Therefore, combined two or more imaging technologies, could compensate limitations of each single imaging modality and improve the performance in medical diagnosis.

**Figure 1:**
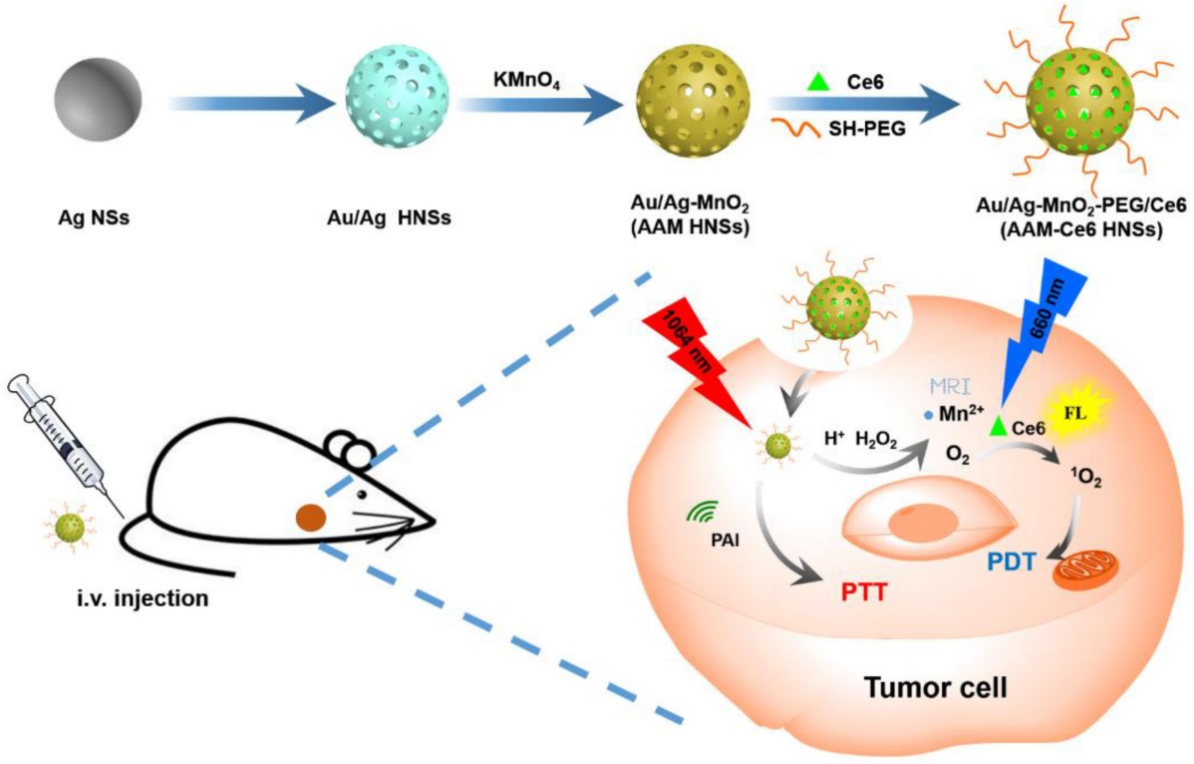
Schematic illustration of the design and synthesis of AAM-Ce6 HNSs for multimodal imaging-guided cancer phototherapy.

In this study, we designed intelligent nanotheranostics based on Au/Ag-MnO_2_ hollow nanospheres (AAM HNSs), which afforded multimodal imaging-guided NIR-II PTT and enhanced PDT therapy. The overall synthetic procedure is schematically illustrated in Figure 1. The Au/Ag alloy HNSs were first synthesized by galvanic replacement reaction according to equation (1), and then the MnO_2_ nanoparticles were deposited on the Au/Ag alloy HNSs by the reaction between Ag and permanganate (KMnO_4_) according to equation (2), using remnant Ag nanospheres (NSs) as the reductant, and finally obtained the AAM HNSs.

3 Ag (s) +AuCl_4_^-^ (aq) → Au (s) +3 AgCl (s) +Cl^-^ (aq) (1)

3 Ag (s) +MnO_4_^-^ (aq) +4 H^+^ → MnO_2_ (s) +3 Ag^+^ +2 H_2_O (2)

Then, SH-PEG was modified on the surface of AAM HNSs by the interaction of sulfhydryl and Au/Ag alloy, which improved the dispersibility and biocompatibility of the HNSs. Next, the PDT photosensitizer Ce6 was loaded into AAM HNSs, benefiting from the hollow interior of the structure, and the AAM-Ce6 HNSs were obtained.

The AAM HNSs exhibit broad absorption at the near infrared (NIR) biological window and remarkable photothermal conversion ability in the NIR-II window. The MnO_2_ nanoparticles on AAM-Ce6 HNSs would decompose in an acidic environment with a large amount of endogenous H_2_O_2_. The generated Mn^2+^ ions could then be used to enhance T_1_-magnetic resonance (MR) imaging contrast for tumor-specific imaging. Simultaneously, endogenous H_2_O_2_ could be decomposed into water and oxygen, catalyzed by AAM-Ce6 HNSs. With the generation of massive oxygen, the tumor hypoxia could be relieved, resulting in the enhanced PDT therapeutic effect. Furthermore, the AAM-Ce6 HNSs displayed excellent fluorescence (FL), photoacoustic (PA), and magnetic resonance (MR) imaging activity. Thus, the AAM-Ce6 HNSs could intelligently respond to TME, afford multimodal imaging-guided NIR-II PTT and enhance the PDT therapy effect, which showed advantages in the development of nanotheranostics.

## Experimental section

### Materials and instrumentation

Silver nitrate (AgNO_3_), ascorbic acid (C_6_H_8_O_6_), sodium hydroxide (NaOH), sodium chloride (NaCl), chloroauric acid trihydrate (HAuCl_4_·3H_2_O), potassium permanganate (KMnO_4_), polyvinyl pyrrolidon (PVP, K30), dimethyl sulfoxide (DMSO) and hydrogen peroxide (H_2_O_2_, 30 %) were obtained from Sinopharm Chemical Reagent Co., Ltd., Shanghai, China. Ce6, 9, 10-anthracenediyl-bis (methylene) dimalonic acid (ABDA) and 2, 7-dichlorodihydrofluorescein diacetate (DCFH-DA) were purchased from Sigma-Aldrich. Thiol group polyethylene glycol (PEG-SH, M_w_~5k) was purchased from Shanghai Yare Biotech, Inc. All reagents were analytical grade and used without further purification.

Cell lines and animals: human cervical cancer cells (HeLa, provided by KeyGEN Biotechnology Co., Ltd.) were cultured in high-glucose DMEM with 10 % FBS at 37 °C in a 5 % CO_2_ atmosphere. Female BALB/c tumor-bearing mice (4-6 weeks) were provided by Jiangsu KeyGEN Biotechnology Co., Ltd. All animal experiments were performed with the permission of the Animal Ethics Committee of Simcere BioTech Corp., Ltd., according to the guidelines approved by Jiangsu Administration of Experimental Animals.

The morphology of various nanoparticles was characterized by HT7700 transmission electron microscopy (TEM). Dynamic light scattering (DLS) studies were conducted using ALV/CSG-3 laser light scattering spectrometers. The UV-vis spectra were analyzed by using a UV3600 UV-vis-NIR spectrophotometer (Shimadzu). Dissolved oxygen was measured by using a portable dissolved oxygen meter (JPBJ-608, INESA Scientific Instrument Co., Ltd, Shanghai, China).

### Synthesis and characterization of AAM-Ce6 HNSs

**Synthesis of silver nanospheres (Ag NSs):** Silver NSs of 50 nm were synthesized from previously adopted literature procedures 46. Briefly, 20 mL of ultrapure water and 85 mg of PVP were added to a round bottom flask and stirred until dissolved. Then, 85 mg of AgNO_3_ was added to the flask. After the AgNO_3_ was completely dissolved, 200 μL of 5 M NaCl solution was added under rapid stirring and stirred for 15 minutes in a dark room to obtain a silver chloride (AgCl) colloid. Another round bottom flask was filled with 2.5 mL of 0.5 M NaOH solution and 20 mL of 50 mM AA solution, and then 2.5 mL of the AgCl colloid solution mentioned above was added dropwise and stirred for 2 hours in the dark to obtain 50 nm Ag NSs.

**Synthesis of the AAM HNSs:** AAM HNSs were synthesized by means of the galvanic replacement reaction between Ag NSs and HAuCl_4_, according to the previously reported method 47. Briefly, the Ag NSs prepared above were diluted into a 100 mL aqueous solution, and then 200 mg of PVP was added. The mixture was heated to boiling for 10 minutes, and then 30 mL of HAuCl_4_ solution (0.2 mM) was added dropwise, while continuing to boil for 10 minutes, and finally cooled to room temperature. The byproduct AgCl was removed by washing with a saturated NaCl solution and then centrifuged and washed with water several times to obtain Au/Ag alloy HNSs. The solution of Au/Ag alloy HNSs prepared above was dropped to 50 mL of KMnO_4_ solution (40 mM) and stirred at room temperature for 30 minutes. Unreacted KMnO_4_ was removed by centrifugation and water washing to obtain the AAM HNSs.

**Synthesis of the AAM-Ce6 HNSs:** 50 mg of SH-PEG was added to 10 mL of AAM HNSs solution (1 mg/mL), stirred at room temperature for 12 hours, and the excess SH-PEG was removed by centrifugation and water washing. Different concentrations of Ce6 were added while stirring at room temperature for 12 hours to obtain AAM-Ce6 HNSs loaded with different amounts of Ce6.

### Measurement of O_2_ and ^1^O_2_ generation* in vitro*

The change of oxygen content in different solutions with time was monitored by the portable dissolved oxygen meter, and the AAM HNSs catalytic performance under different conditions was investigated. Briefly, PBS solutions containing H_2_O_2_ (100 μM) with different pH values were added to a sealed bag, and then the AAM HNSs sample solution (100 μg/mL) was added while maintaining moderate shaking. The value of dissolved O_2_ was detected by the sensor and recorded at predetermined time points.

Following previously reported methods 38, 48, ^1^O_2_ production was detected by UV-vis spectroscopy using ABDA as the ^1^O_2_ indicator. Briefly, 100 μL of ABDA solution (1 mg/mL) was thoroughly mixed with AAM-Ce6 HNSs in PBS solutions with different pH values. H_2_O_2_ was added to the above solutions at a final concentration of 100 μM. The mixtures were then irradiated with a 660 nm laser with a power density of 100 mW/cm^2^ for 10 minutes. The ^1^O_2_ content was determined by measuring the change in absorbance of ABDA at 378 nm for different irradiation times, and ABDA in PBS (pH 5.5) under the same laser irradiation was set as a control.

### Ce6 loading and release *in vitro*

Different concentrations of Ce6 solutions (in DMSO) were added to AAM HNSs solutions (0.25 mg/mL) and stirred for 12 hours at room temperature. The excess Ce6 was removed by centrifugation with DMSO and water mixture. The amount of Ce6 loaded was quantitatively evaluated by the absorbance at 665 nm.

The pH response release profile of Ce6 in AAM-Ce6 HNSs was estimated by the previously reported method 49. Briefly, the determination of Ce6 content in AAM-Ce6 HNSs diluted with 10 mL of PBS (pH 5.5 or 7.4). Then, a 1 mL aliquot of the solution was withdrawn and centrifuged to collect the supernatant at different time points. The absorbance of the supernatant at 665 nm was recorded, and the amount of Ce6 was calculated as mentioned above by fitting the Ce6 standard UV absorption curve.

### Photothermal imaging and photothermal conversion efficiency

AAM-Ce6 HNSs solutions with different concentrations were placed in the quartz cuvette, and then each group was irradiated for 10 minutes at 808 nm or 1064 nm laser. The variations of temperature over irradiation time were recorded. A sample solution with a concentration of 40 μg/mL was used to calculate the photothermal conversion efficiency. Briefly, the sample solution was irradiated at 808 nm or 1064 nm for 10 minutes, then the laser was turned off, and the solution allowed to cool naturally for 10 minutes. The photothermal conversion efficiency was calculated using formula (3) according to the previously reported method 50, please refer to the supplementary file for details.


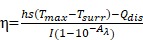
(3)

To evaluate the photothermal stability, the sample solutions with a concentration of 40 μg/mL were irradiated with an 808 nm or 1064 nm laser. The temperature profiles of the sample solutions were recorded for four successive cycles of heating/cooling processes under 808 nm or 1064 nm. To evaluate the penetration ability of different lasers to tissues, the samples were covered with chicken breasts of different thicknesses and then irradiated at 808 nm or 1064 nm (1 W/cm^2^). All experiments were monitored and recorded by the FLIR E50 infrared camera.

### Cellular uptake, hypoxia analysis and intracellular ROS detection *in vitro*

HeLa cells were seeded in confocal dishes with a density of approximately 1×10^5^ cells per dish and incubated for 24 hours to allow cells to attach. AAM-Ce6 HNSs (100 μg/mL) were added to the culture dishes, and the cells were incubated at 37 °C for different time points and then washed three times with PBS. The nuclei were stained with 4', 6'-diamidino-2-phenylindole (DAPI) solution (5 μg/mL). Cellular imaging of HeLa cells was performed by an Olympus FV 1000 laser confocal scanning microscope, and fluorescence emission of Ce6 was collected under 635 nm laser excitation.

For hypoxia detection, HeLa cells from different treatment groups were incubated in N_2_ environment for different times. The cellular hypoxia was analyzed by confocal scanning microscopy using the ROS-ID Hypoxia/Oxidative stress detection kit (Enzo Life Sciences) according to the manufacturer's instructions (for fluorescence signal of hypoxia, excitation filter: 420 nm, emission filter: 600-700 nm).

Intracellular ROS generation was monitored by a reactive oxygen species assay kit based on DCFH-DA. The cells were incubated with AAM-Ce6 HNSs (100 μg/mL) at 37 °C and 5 % CO_2_ for 4 hours and washed three times with PBS. Then, the cells were incubated with DCFH-DA (10 μM) for 10 minutes to detect the intracellular singlet oxygen generation followed by irradiation with a 660 nm laser (100 mW/cm^2^, 5 min). Fluorescence imaging (FL) of DCF was performed using confocal microscopy (excitation filter: 488 nm, emission filter: 525 nm).

### Cytotoxicity assay

HeLa cells were seeded in 96-well plates with a density of approximately 1×10^5^ cells per well and incubated for 24 hours to allow cells to attach. For dark toxicity, different concentrations of AAM HNSs and AAM-Ce6 HNSs were added to the wells and cultured in the dark for 12 hours. For phototoxicity, different concentrations of AAM-Ce6 HNSs were added to the wells and cultured for 12 hours. Then, the cells were washed three times with PBS, and the cells in each well were exposed to 1064 nm, 808 nm (for PTT, 1 W/cm^2^) or 660 nm (for PDT, 100 mW/cm^2^) lasers for 5 minutes. After another 4 hours of incubation, the relative cell viabilities were measured by the methyl thiazolyl tetrazolium (MTT) assay.

### *In vitro* and *in vivo* FL, PA, and MR imaging

For FL imaging, free Ce6 and 150 μL AAM-Ce6 HNSs solution (1 mg/mL, equal amount of Ce6) were intravenously (i.v.) injected into HeLa tumor-bearing mice through their tail vein. At predetermined time points, the FL images were acquired by the IVIS Lumina K *in vivo* fluorescence imaging system (PerkinElmer, USA), excitation filter: 660 nm, emission filter: 710 nm. The mice were sacrificed after 24 hours, and *ex vivo* imaging was performed on the excised tumors and organs.

For *in vitro* MRI, an ICON preclinical compact magnetic resonance imager was used to test AAM-Ce6 HNSs after treatment with H_2_O_2_ solution at different pH values. The relaxation rate r_1_ (1/T_1_) was then calculated from the T_1_ values of different Mn^2+^ concentrations. For *in vivo* MRI, 150 μL of the AAM-Ce6 HNSs solution (1 mg/mL) was i.v. injected into HeLa tumor-bearing mice through their tail vein, MR images of the tumor were acquired at different time points postinjection.

For *in vitro* PAI, a preclinical photoacoustic computerized tomography scanner (Endra Nexus 128, USA) was used to detect the PAI signals of different concentrations of AAM-Ce6 HNSs solution at an 850 nm laser. For *in vivo* PAI, HeLa tumor-bearing mice were i.v. injected with 150 μL AAM-Ce6 HNSs solution (1 mg/mL) via rapid injection through their tail vein. PA images of the tumor were acquired at different time points postinjection.

### Effect of AAM-Ce6 HNSs on tumor oxygenation and PDT/PTT treatment *in vivo*

To confirm that the AAM-Ce6 HNSs have the ability to improve the tumor hypoxic environment, a hypoxia probe immunofluorescence assay (pimonidazole, Hypoxyprobe-1 plus kit, Hypoxyprobe Inc.) was performed to examine tumor slices extracted after i.v. injection of different nanomaterials. HeLa tumor-bearing mice were i.v. injected with PBS, Au/Ag HNSs or AAM-Ce6 HNSs (150 µL, 1 mg/mL), respectively. After 12 hours, tumor-bearing mice were surgically excised 90 min after intraperitoneal injection with pimonidazole hydrochloride (60 mg/kg). Frozen sections of the tumors were created by optimum cutting temperature compound (Sakura Finetek) for immunofluorescence staining. The tumor sections were incubated with mouse anti-pimonidazole antibody (dilution 1:100, Hypoxyprobe Inc.) and Alex 488-conjugated goat anti-mouse secondary antibody (dilution 1:200, Jackson Inc.) according to the manufacturer's instructions. Tumor blood vessels were stained by rat anti-CD31 mouse monoclonal antibody (dilution 1:200, Biolegend) and Rhodamine-conjugated donkey anti-rat secondary antibody (dilution 1:200, Jackson). Cell nuclei were stained with DAPI, the slices were imaged using CLSM.

HeLa tumor-bearing mice were randomly divided into five groups when the tumor reached approximately 150 cm^3^ (four in each group): (1) saline (for control), (2) AAM-Ce6 HNSs, (3) AAM-Ce6 HNSs +660 nm, (4) AAM-Ce6 HNSs +1064 nm, (5) AAM-Ce6 HNSs +660 & 1064 nm. The mice in groups (2) ~ (5) were i.v. injected with 150 µL AAM-Ce6 HNSs solution (1 mg/ mL). After 12 hours, the laser irradiation groups were irradiated with a 1064 nm laser (1 W/cm^2^, 10 min) for PTT and a 660 nm laser (100 mW/cm^2^, 10 min) for PDT. The curve of temperature heating and cooling of the tumor site was obtained, and the mice continued to be raised for 15 days after initial treatment. Tumor volume and body weight of each mouse were recorded every two days after treatment. The treated mice were sacrificed on day 15, and tumors were excised and weighed. The inhibition rate (IR) was calculated as follows: IR (%) = [(x-y)/x] ×100 %, where x and y represent the mean tumor weight of the control and treatment groups, respectively. The tumors and main organs (heart, liver, spleen, lungs and kidneys) were collected and examined by hematoxylin and eosin (H&E) staining to assess the biocompatibility of the AAM-Ce6 HNSs.

## Results and Discussion

### Synthesis and characterization of AAM-Ce6 HNSs

**Figure 2:**
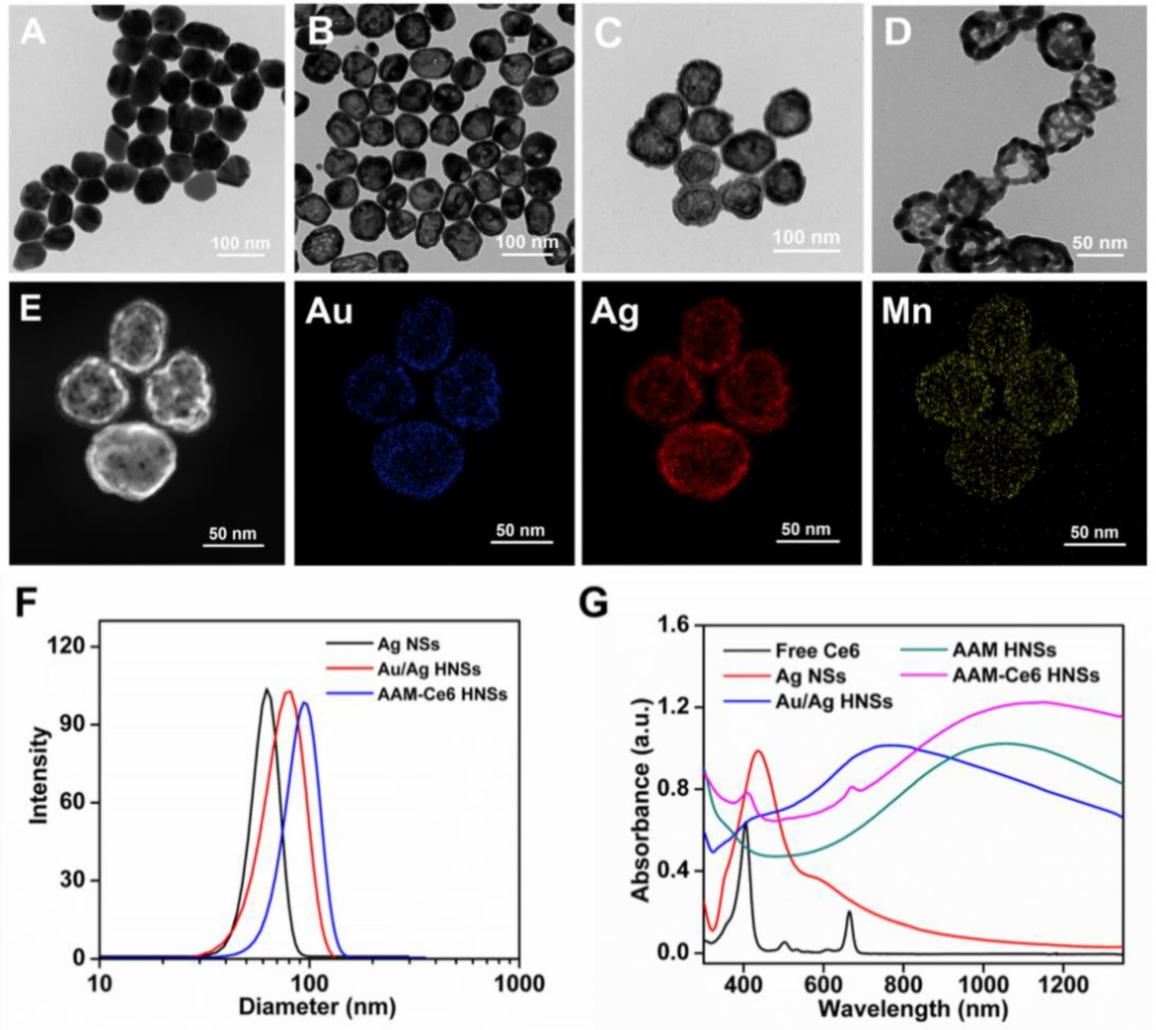
TEM images of (A) Ag NSs, (B) Au/Ag HNSs and (C) AAM-Ce6 HNSs, and (D) AAM-Ce6 HNSs after dissolved in a PBS solution containing 100 μM H_2_O_2_, pH 5.5. (E) HAADF-STEM images and Au, Ag and Mn elemental mapping of AAM-Ce6 HNSs. (F) Hydrodynamic diameter of Ag NSs, Au/Ag HNSs and AAM-Ce6 HNSs measured by DLS, the polydispersity are 0.185, 0.231, 0.206 respectively. (G) UV-vis-NIR spectra of free Ce6, Ag NSs, Au/Ag HNSs, AAM HNSs and AAM-Ce6 HNSs.

The synthesis schematic diagram of AAM-Ce6 HNSs is shown in Figure 1. First, Au/Ag alloy HNSs were synthesized by a galvanic replacement reaction between HAuCl_4_ and Ag NSs using Ag NSs as a self-sacrifice template. Subsequently, the solution of Au/Ag alloy HNSs prepared above was added dropwise to a KMnO_4_ solution to fabricate AAM HNSs. Then, SH-PEG was attached to the AAM HNSs surface by the interaction of the sulfhydryl group with Au/Ag alloy, and then Ce6 was loaded into the hollow structure of AAM HNSs. The transmission electron microscope (TEM) images of various nanostructures are shown in Figure 2, Figures 2A-D show TEM images of Ag NSs, Au/Ag HNSs, AAM-Ce6 HNSs and broken AAM-Ce6 HNSs (dissolved in PBS solution for half an hour containing 100 μM H_2_O_2_, pH 5.5), respectively. From the TEM images, it can be seen that the Ag NSs, Au/Ag HNSs and AAM-Ce6 HNSs showed quasi-spherical morphology and uniform size distribution. In addition, the Au/Ag HNSs and AAM-Ce6 HNSs show an interior hollow nanostructure, which makes it possible to be a drug carrier. When AAM-Ce6 HNSs were dissolved in acidic PBS solution containing H_2_O_2_, the MnO_2_ decomposed by reaction with H^+^ and H_2_O_2_, generating Mn^2+^ ions (and release of a payload), which indicates the sensitive response of AAM-Ce6 HNSs to TME. The high-angle annular dark-field scanning transmission electron microscopy energy-dispersive X-ray spectroscopy (HAADF-STEM-EDX) mapping (Figure 2E) clearly revealed that Au, Ag and Mn elements were distributed on the HNSs, proving the successful preparation of AAM HNSs. The diameter of the AAM-Ce6 HNSs was approximately 95 nm, as measured by dynamic light scattering (DLS) (Figure 2F), with a uniform size distribution, which is consistent with the TEM results. The hydrodynamic diameters of AAM-Ce6 HNSs dispersed in water, PBS and FBS for 7 days were measured by DLS, which proved the good stability of AAM-Ce6 HNSs in different solutions (Figure S2A). With the modification of SH-PEG, the AAM-Ce6 HNSs could be well dispersed in different solutions without aggregation (Figure S1A). The changes of zeta potentials for nanoparticles obtained at different steps of fabrication (Figure S2B). The UV-vis-NIR spectra of free Ce6, AAM HNSs, and AAM-Ce6 HNSs are shown in Figure 2G. A broad distinctive peak at approximately 300-400 nm could be attributed to the surface plasmon band of MnO_2_ 51. The characteristic peaks of Ce6 (405 nm, 665 nm) could also be observed in the absorption spectra of AAM-Ce6 HNSs, indicating the successful loading of Ce6 in AAM HNSs. The broad and strong absorption peak at approximately 1100 nm could be attributed to the surface plasmon resonance absorption of Ag/Au HNSs. In addition, AAM-Ce6 HNSs had specific fluorescence emission when excited at the wavelength of 420 nm, which could be further used for fluorescence observation (Figure S3).

As mentioned above, the synthesized AAM-Ce6 HNSs showed a strong and broad absorption peak at approximately 1100 nm in the NIR-II window. The photothermal effect of AAM-Ce6 HNSs *in vitro* was evaluated with 1064 nm and 808 nm lasers. As shown in Figures S5A and S5B, the temperature of AAM-Ce6 HNSs increased rapidly over time at the beginning, reaching a plateau after 5 minutes of laser irradiation. However, there was no appreciable temperature change for the control sample (PBS solution), which proved the excellent photothermal properties of AAM-Ce6 HNSs. In addition, the temperature under the 1064 nm laser irradiation was slightly higher than that under 808 nm laser irradiation, which could be attributed to the higher absorption intensity of AAM-Ce6 HNSs at 1064 nm (Figure S4). According to the reported calculation method, the photothermal conversion efficiency (η) of AAM-Ce6 HNSs was calculated to be 52.5 % at 1064 nm and 44.0 % at 808 nm (Figures S5C and S5D). It is also noteworthy that the photothermal conversion efficiency under 1064 nm laser irradiation was even higher than that of reported Au/Ag alloy double nanoshells 9, copper sulfide nanoparticles and gold-based hybrid nanomaterials 5, 8, 52. The photothermal heating curves of AAM-Ce6 HNSs remained unchanged after four cycles (Figure S6), implying the good photothermal stability of AAM-Ce6 HNSs.

**Figure 3:**
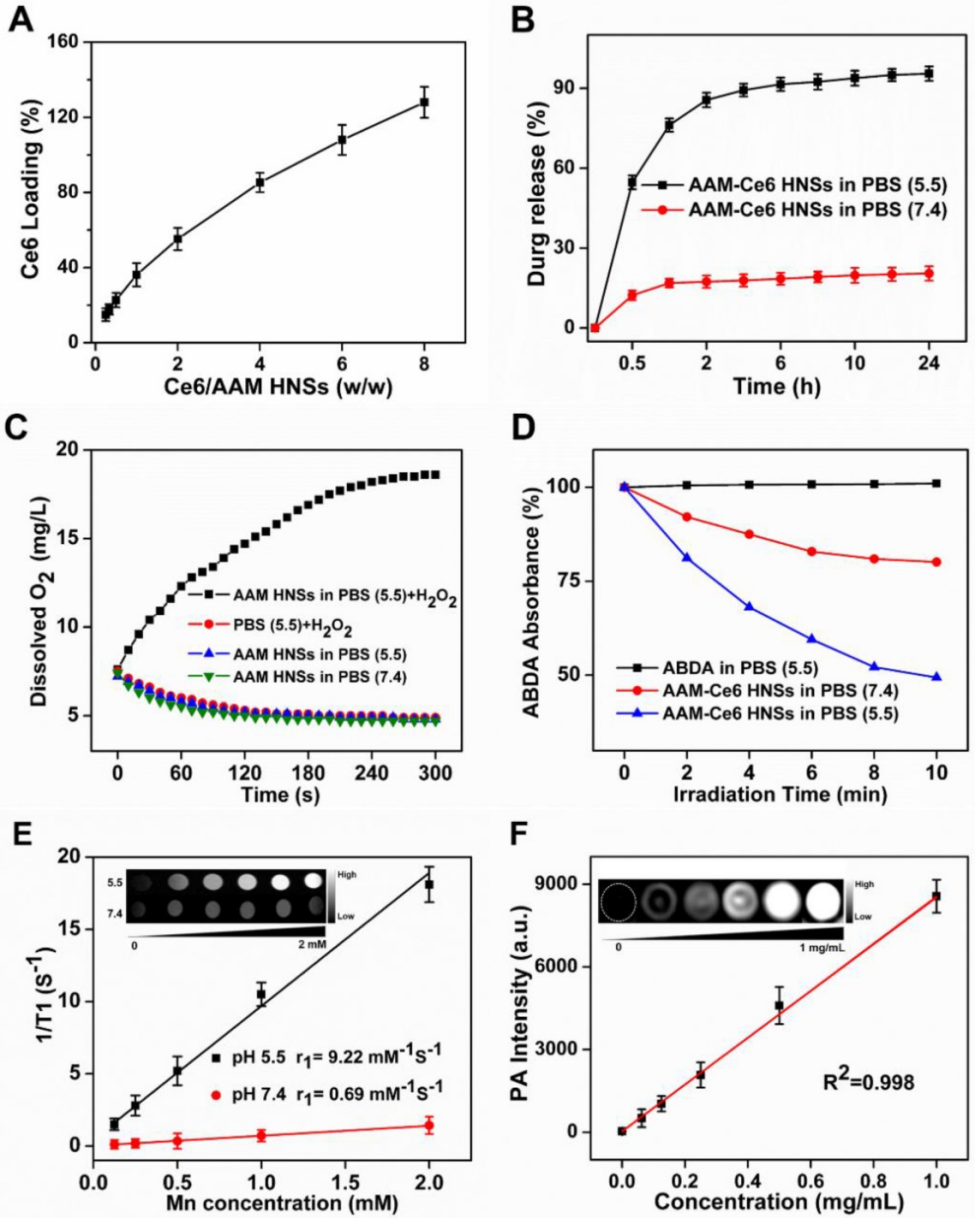
(A) Ce6 loading in AAM HNSs at different feeding ratios. (B) Time-dependent release of Ce6 from AAM-Ce6 HNSs at different pH values. (C) Dissolved oxygen amount in AAM HNSs solutions (100 μg/mL) with/without H_2_O_2_ (100 μM) at different pH values. (D) The generation of ^1^O_2_ determined by the change in ABDA absorbance in different reaction systems. (E) T_1_ relaxation rate of AAM-Ce6 HNSs in different concentrations at different pH values. Inset is the T_1_-weighted MR imaging of AAM-Ce6 HNSs in different pH PBS solutions. (F) PAI signal intensity of AAM-Ce6 HNSs at different concentrations. Inset is the PAI signal images of AAM-Ce6 HNSs at different concentrations.

It is well known that the NIR-II window has lower tissue absorption and scattering, so using the NIR-II window can achieve a larger tissue penetration depth than the NIR-I window. Chicken breasts with different thicknesses were used as model tissue to explore the photothermal effect of AAM-Ce6 HNSs in the NIR-II windows and NIR-I windows (Figure S7). The temperature changes of AAM-Ce6 HNSs solution covered by chicken breasts with different thicknesses were recorded under 1064 nm and 808 nm laser irradiation. The results showed that AAM-Ce6 HNSs could reach a relatively higher temperature at the 1064 nm laser than at the 808 nm laser, regardless of the thickness of the chicken breast (Figure S8). For instance, the photothermal attenuation of the 1064 nm laser was 38.6 % when covered with 6 mm of chicken breast, which was much lower than that of the 808 nm laser (46.4 %) (Table S1). The results suggested that with the high absorption in the NIR-II window, a better tissue penetration depth and PTT therapeutic effect for a deep tumor could be obtained. Because of the interior hollow structure and TME-responsive behavior of the synthesized AAM HNSs, it could be an ideal intelligent theranostic nanoplatform. The loading amount of Ce6 was determined by UV-vis spectra. When the weight ratio of Ce6 and AAM HNSs increased, the loading amount of Ce6 increased; when the ratio was 8:1, it reached a relatively high loading content of approximately 125 % (Ce: AAM HNSs, w/w), which was used for subsequent experiments (Figure 3A). To study the release behaviors, the AAM-Ce6 HNSs were dissolved in PBS with different pH values. The release curves of Ce6 were obtained according to the absorption value of supernatant at 665 nm at different time points (Figure 3B). The release rate of Ce6 was faster in PBS solution at pH 5.5 than in PBS solution at pH 7.4. After 24 hours of incubation in the acidic solution, approximately 95.5 % of the loaded Ce6 was released from the AAM HNSs, triggered by the decomposition of MnO_2_ in an acidic environment.

It is well recognized that MnO_2_ can promote the decomposition of H_2_O_2_ into water and oxygen. In addition, under acidic conditions, MnO_2_ can be used as both a catalyst and reactant, and Mn^2+^ and O_2_ can be produced by the consumption of H_2_O_2_ and H^+^ in the tumor 37, 53. The oxygen generation capacity of AAM HNSs in different systems was investigated by an oxygen sensor. Considering the existence of endogenous H_2_O_2_ inside most types of solid tumors, with concentrations in the range of 10-100 μM 54, the catalytic ability of AAM HNSs was investigated in the different solutions. As shown in Figure 3C, due to the catalytic ability of MnO_2_ in AAM HNSs, oxygen could be generated in the presence of H_2_O_2_ (100 μM, pH 5.5), and no oxygen was generated in the system of AAM HNSs or H_2_O_2_ alone. In addition, a large number of oxygen bubbles could be observed after adding AAM HNSs (100 μg/mL) to the H_2_O_2_ solution (10 mM, pH 5.5), indicating that AAM HNSs had excellent ability to initiate H_2_O_2_ decomposition and generation of oxygen (Figure S1B).

The AAM-Ce6 HNSs has the ability to promote the decomposition of H_2_O_2_ and generate O_2_, which would accelerate ^1^O_2_ production. The ^1^O_2_ production of AAM-Ce6 HNSs under laser irradiation in different solutions was measured by ^1^O_2_ indicator, ABDA. As expected, in the presence of AAM-Ce6 HNSs and H_2_O_2_ at pH 5.5, the absorbance of ABDA changed significantly under 660 nm laser irradiation, indicating a large amount of ^1^O_2_ production (Figure 3D). However, the absorbance of ABDA changed slightly in the case of AAM-Ce6 HNSs in the solution at pH 7.4, showing a lower production of ^1^O_2_. This proved that the AAM-Ce6 HNSs could promote the decomposition of H_2_O_2_ and generate O_2_, which relieved tumor hypoxia and enhanced the PDT effects (Figure S9). On the other hand, ^1^O_2_ production can also be improved by increasing the temperature 55. The production of ^1^O_2_ was investigated by a singlet oxygen sensor green probe (SOSG), which can emit fluorescent signals when reacting with ^1^O_2_ 56. As shown in Figure S10, the SOSG fluorescent signal of AAM-Ce6 HNSs under irradiation with 660 and 1064 nm lasers was much stronger than that under irradiation with 660 nm laser alone. In addition, the fluorescent signal was stronger in the presence of H_2_O_2_ at pH 5.5 than that without H_2_O_2_, which could be ascribed to the O_2_ generation in this environment. These results indicated that AAM-Ce6 HNSs could increase the PDT effect of Ce6 by photohyperthermia and that TME-triggered O_2_ generation.

As mentioned above, MnO_2_ nanoparticles could be decomposed into Mn^2+^ in an acidic environment, generating Mn^2+^ ions that could be a T_1_-shortening agent in MRI. The enhanced T_1_-weighted MRI property of AAM-Ce6 HNSs was investigated in a simulated TME. As displayed in Figure 3E, compared to that of neutral solution (PBS, pH 7.4) containing H_2_O_2_ (100 μM), the MRI images in acidic environment (PBS, pH 5.5) showed obvious enhanced whitening effects. Furthermore, the relaxation rate (r_1_) in acidic environments was calculated to be 9.22 mM^-1^s^-1^, which was much higher than commercial magnetic resonance contrast agents (Magnevist^®^, r_1_≈3.40 mM^-1^s^-1^) 57. This could be attributed to the TME-triggered generation of massive paramagnetic Mn^2+^ ions.

Since there is broad, strong absorption in the NIR window, AAM-Ce6 HNSs could be used as a photothermal agent. Light absorption of AAM-Ce6 HNSs created a thermally induced pressure jump that launched ultrasonic waves, and the ultrasonic waves could be received by acoustic detectors from images 58. The PAI signals of AAM-Ce6 HNSs at different concentrations were investigated. As shown in Figure 3F, as the AAM-Ce6 HNSs concentration increased, the PAI signal increased gradually, and the intensity of PAI signals was linear with the concentrations of AAM-Ce6 HNSs. This result indicated that the AAM-Ce6 HNSs could be applied to PAI *in vivo*.

### Cellular experiments and antitumor efficacy of PTT and PDT *in vitro*

**Figure 4:**
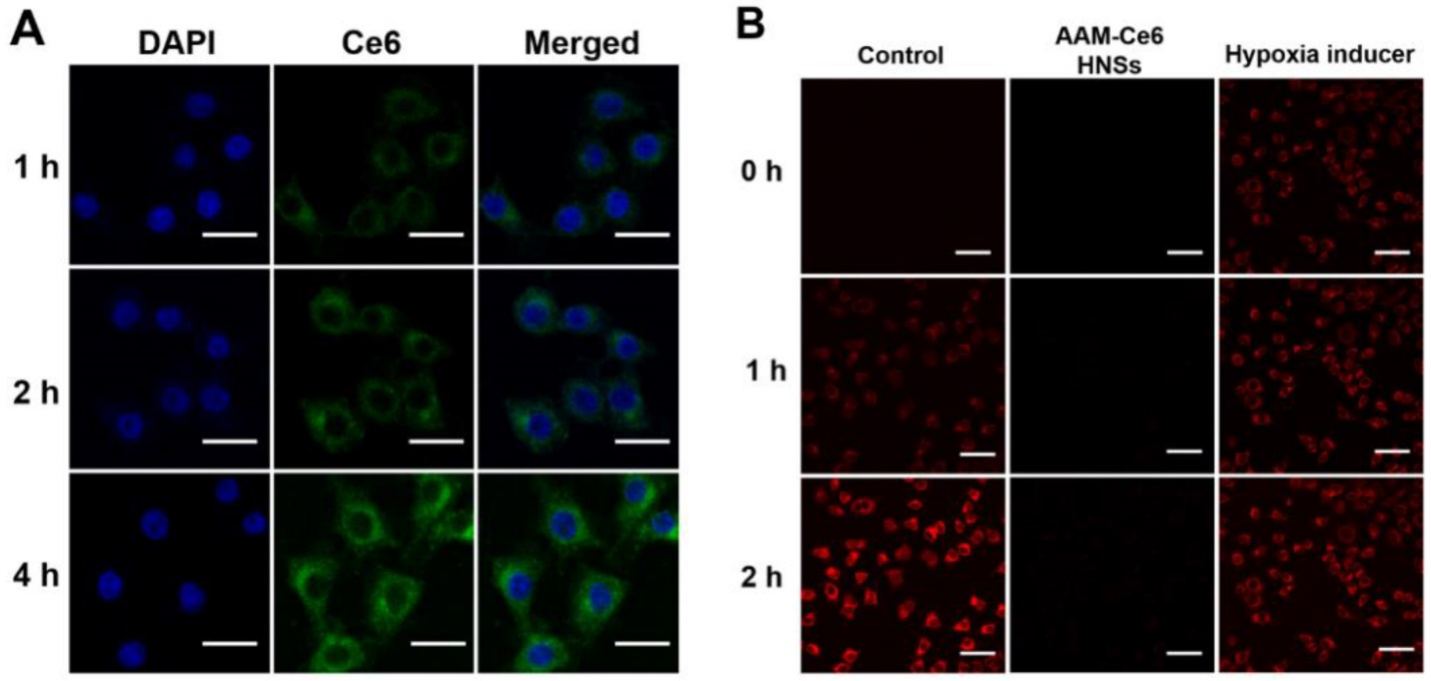
(A) Confocal fluorescence images of HeLa cells incubated with AAM-Ce6 HNSs at different time points. The scale bar was 20 μm. (B) Intracellular fluorescence detection of HeLa cells with hypoxia detection probes in different treatment groups. The scale bar was 50 μm.

The cellular uptake of AAM-Ce6 HNSs was investigated by incubating HeLa cells with HNSs for different times, and FL imaging of Ce6 in the cells was performed by confocal microscopy. As shown in Figure 4A, the fluorescence of Ce6 mainly existed in the cytoplasm and increased gradually with incubation time, which indicated that Ce6 was effectively taken up by cells. In addition, this implied that the strong fluorescent signal of Ce6 could be used for FL imaging *in vivo*. HeLa cells from different treatment groups were incubated in an N_2_ environment for 2 hours, and the cellular hypoxia was analyzed by hypoxia detection kits. As shown in Figure 4B, compared with the control group, the hypoxic fluorescent signal of cells treated with AAM-Ce6 HNSs was significantly reduced, showing a strong oxygen generating ability. This result indicated that the hypoxic state of tumor cells could be effectively alleviated by the presence of AAM-Ce6 HNSs, thereby enhancing the therapeutic effect of PDT.

**Figure 5:**
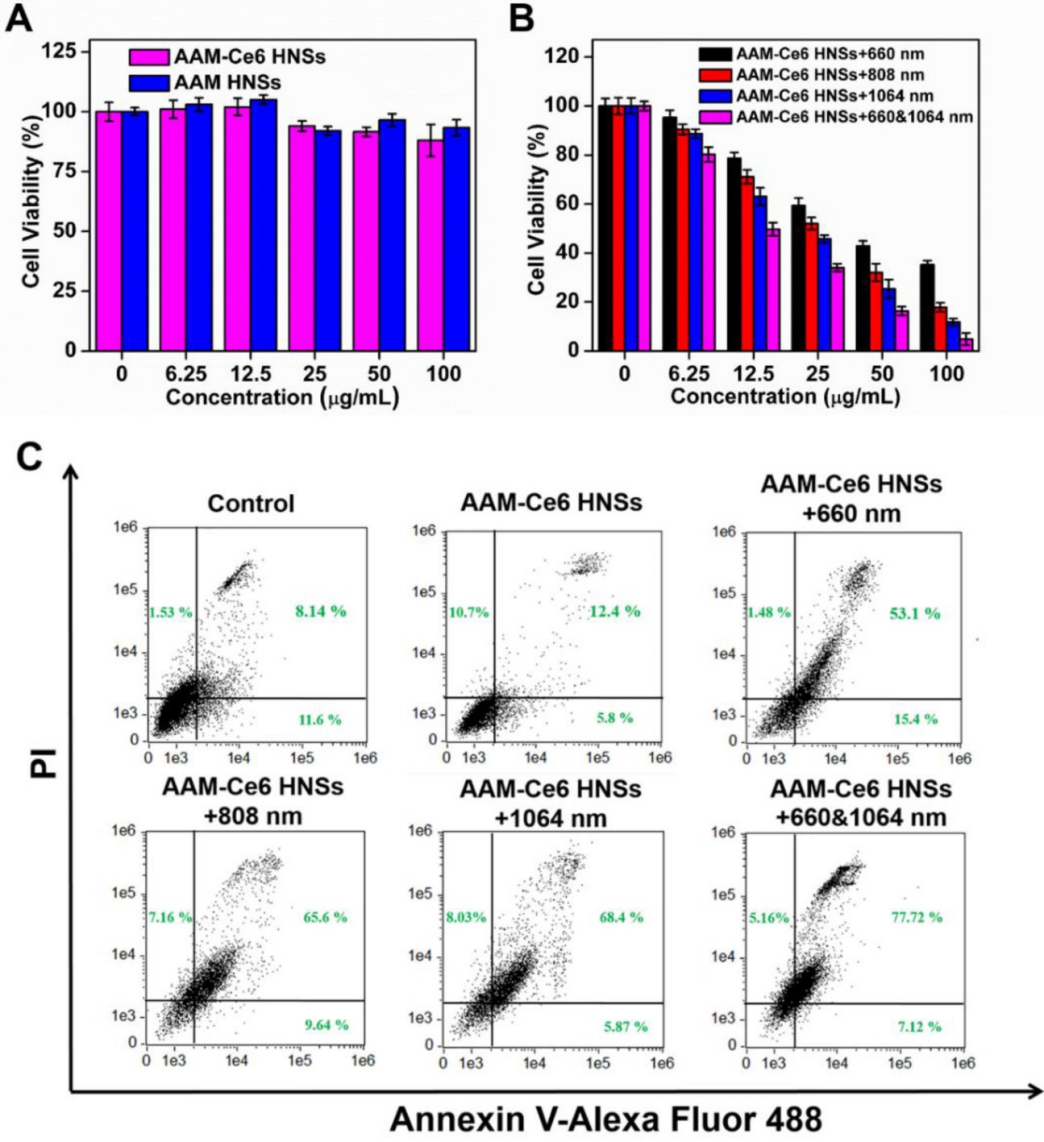
(A) Relative viabilities of HeLa cells after incubation with different concentrations of AAM HNSs and AAM-Ce6 HNSs in the dark for 24 hours. (B) Relative viability of HeLa cells after incubation with different concentrations of AAM-Ce6 HNSs under 660 nm laser irradiation for PDT (100 mW/cm^2^, 5 min), 808 nm or 1064 nm laser irradiation for PTT (1 W/cm^2^, 5 min). (C) Flow cytometry analysis of HeLa cells incubated with AAM-Ce6 HNSs (100 μg/mL) under 660 nm laser irradiation for PDT (100 mW/cm^2^, 5 min), 808 nm or 1064 nm laser irradiation for PTT (1 W/cm^2^, 5 min).

Inspired by the excellent uptake of AAM-Ce6 HNSs by cells, standard MTT assays were performed to examine the cell dark cytotoxicity of AAM HNSs and AAM-Ce6 HNSs. Even at a high dosage (100 μg/ mL), AAM-Ce6 HNSs showed no significant cytotoxicity, and the cell survival rate was higher than 80 %, indicating the good biocompatibility of AAM-Ce6 HNSs (Figure 5A). Subsequently, to evaluate the PDT and PTT therapeutic effects *in vitro,* the cells were incubated with different concentrations of AAM-Ce6 HNSs and were irradiated with 660 nm laser (100 mW/cm^2^, 5 min) for PDT and 808 nm or 1064 nm laser (1 W/cm^2^, 5 min) for PTT.

The results showed that the relative viability of the cells decreased with increasing sample concentrations (Figure 5B), indicating that AAM-Ce6 HNSs had a good concentration-dependent therapeutic effect on PDT and PTT. The photothermal treatment effect of the 1064 nm laser was significantly stronger than that of the 808 nm laser. However, as shown in Figure 5A, the cells showed high viability without light, indicating that the phototherapy effect of AAM-Ce6 HNSs could be controlled by light excitation, which also provided the possibility of selective treatment for tumor cells and avoiding toxic side effects. In addition, with two kinds of simultaneous laser irradiation (660 nm & 1064 nm), the antitumor effect was the best, indicating the great effect of PTT combined PDT on tumor cells *in vivo*. To further confirm the therapeutic effect of AAM-Ce6 HNSs, the apoptosis and necrosis of different treated cell samples were detected by flow cytometry using PI and Annexin V-FITC staining. As shown in Figure 5C, the control and AAM-Ce6 HNSs groups showed lower cell death rates than the laser treatment groups. The cells treated with AAM-Ce6 HNSs irradiated under 660 nm and 1064 nm lasers showed a considerable increase in the apoptosis ratio, which was similar to the result of the MTT assay.

**Figure 6:**
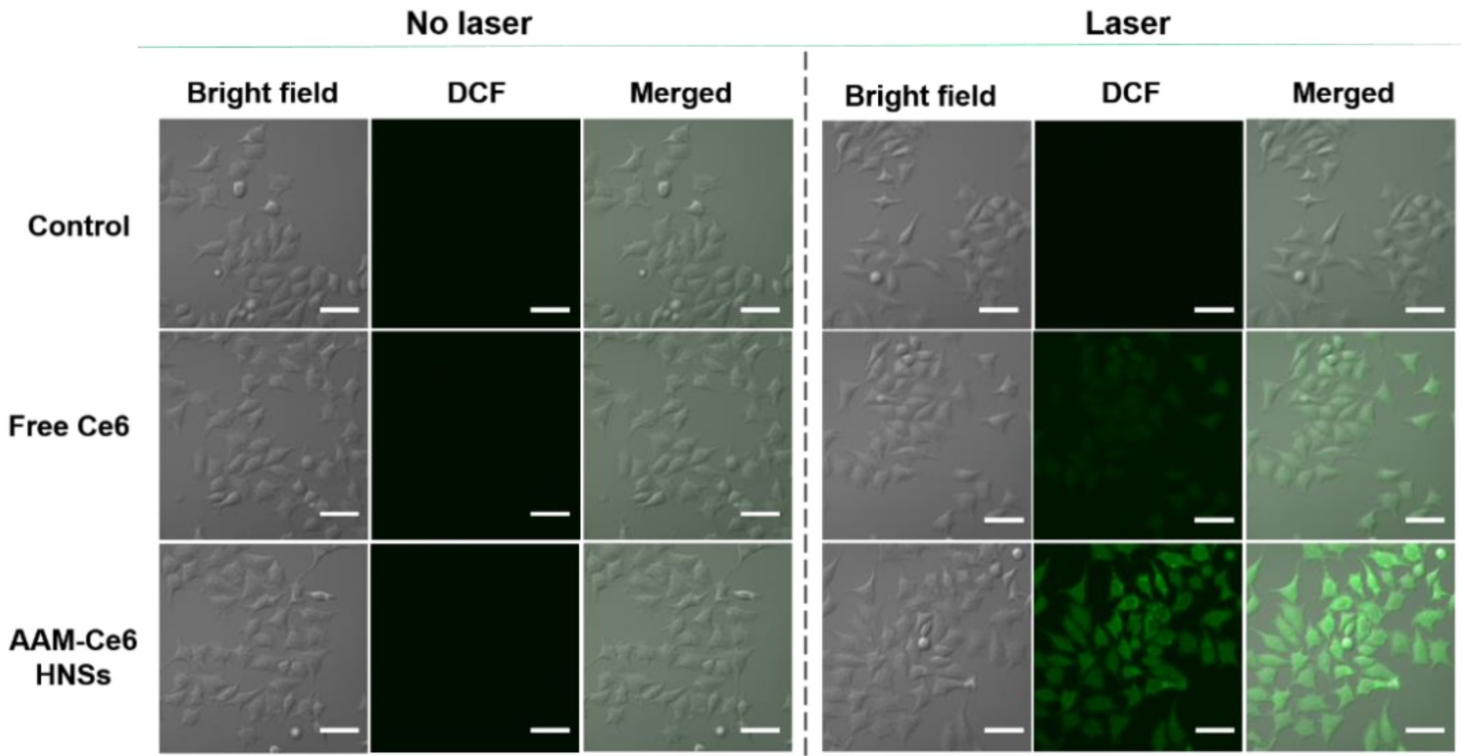
Confocal images of ^1^O_2_ generation by DCFH-DA staining in cells (incubated with cell medium as control). The scale bar is 60 μm.

As described above, AAM-Ce6 HNSs could be efficiently taken up by tumor cells and release Ce6 in TME. HeLa cells were cultured under different conditions, and the ^1^O_2_ generated was measured by the fluorescent indicator DCFH-DA. DCFH-DA is a non-fluorescent molecule that can be passively diffused into cells and turn on bright green fluorescent signals by oxidizing intracellular ^1^O_2_. The fluorescent signal generated from oxidized DCF can be directly observed by confocal microscopy under 488 nm laser irradiation. As shown in Figure 6, the control group did not show a fluorescent signal with or without laser irradiation. However, in the case of the AAM-Ce6 HNSs group, a bright green fluorescence signal could be observed after 660 nm laser irradiation, even stronger than that of the free Ce6 group (with an equal amount of Ce6). This was mainly because MnO_2_ could promote the decomposition of intracellular H_2_O_2_, produce O_2_ in the TME, and enhance the generation of ^1^O_2_.

### *In vivo* FL, MR and PA multimodal imaging with AAM-Ce6 HNSs

**Figure 7:**
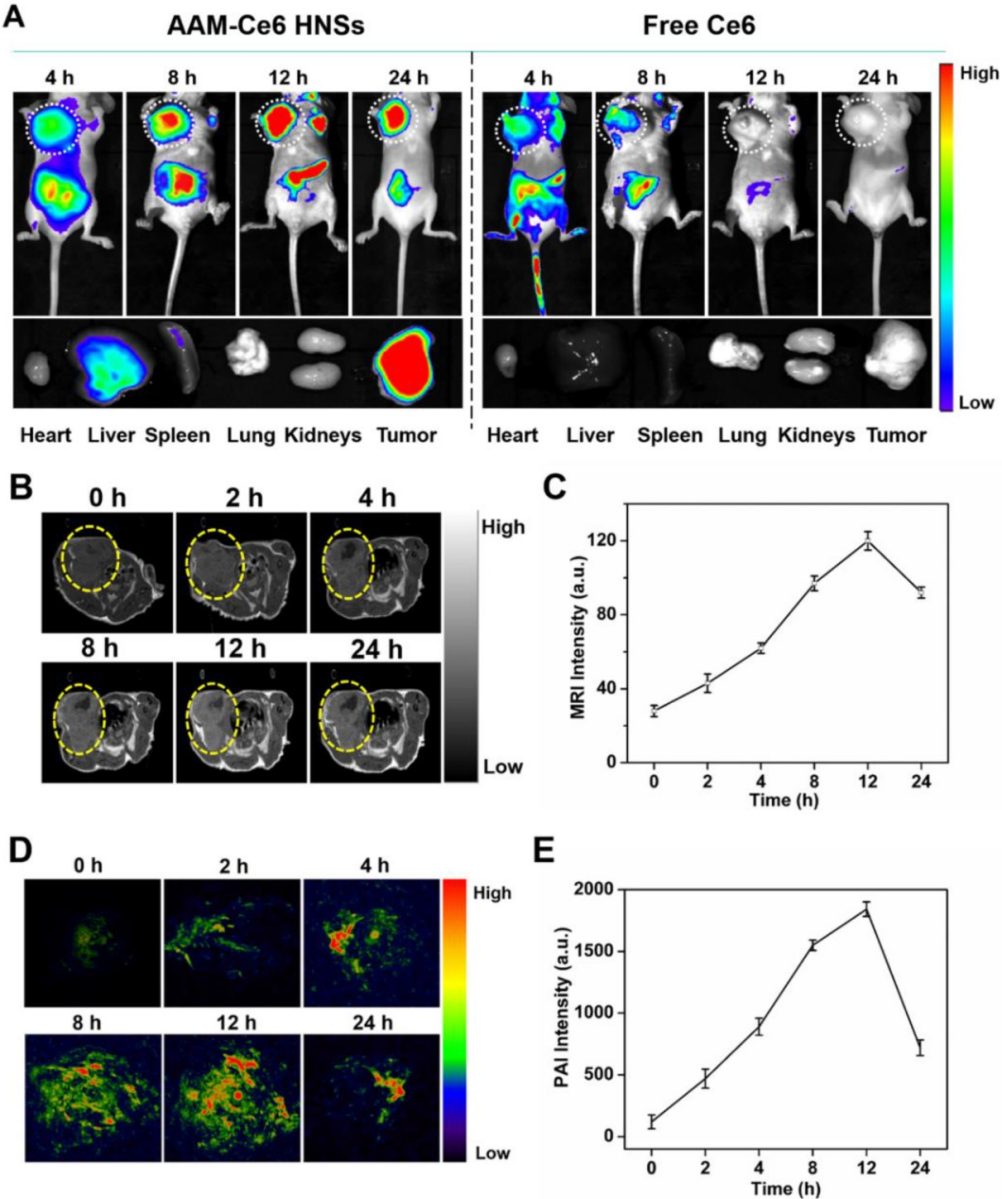
*In vivo* and *ex vivo* imaging with AAM-Ce6 HNSs. (A) FL images of HeLa tumor-bearing mice post i.v. injection of free Ce6 and AAM-Ce6 HNSs solutions, the bottom panel shows *ex vivo* images of isolated organs and tumors examined 24 hours after i.v. injection. (B) T_1_-weighted MRI of HeLa tumor-bearing mice at different time points and (C) MRI signal intensity change postinjection with AAM-Ce6 HNSs. (D) PAI of HeLa tumor-bearing mice at different time points and (E) PAI signal intensity change postinjection with AAM-Ce6 HNSs.

For FL imaging, HeLa tumor-bearing mice were i.v. injected with free Ce6 (as the control) and AAM-Ce6 HNSs solutions (with equal amount of Ce6) through the caudal vein. The fluorescence images were acquired in an IVIS Lumina III imaging system at predetermined time points. According to the FL images, AAM-Ce6 HNSs showed significant tumor accumulation, and the FL intensity reached a maximum after 12 hours i.v. injection (Figure 7A). Moreover, the FL signals of tumor tissues were significantly different from the surrounding normal tissues and remained strong even after 24 hours, indicating that AAM-Ce6 HNSs could passively target tumor tissues and show a longer metabolic time. In contrast, free Ce6 showed relatively weak FL signals in tumor tissues and almost disappeared after 8 hours, indicating that free Ce6 could not accumulate in tumor tissues and would be rapidly excreted from the body. The tumor-bearing mice were sacrificed, and *ex vivo* fluorescence images of the tumor and major organs were obtained 24 hours after injection (Figure 7A bottom panel). A small accumulation of AAM-Ce6 HNSs was observed in the liver. However, a large amount was enriched in the tumor tissue. The FL imaging capability of AAM-Ce6 HNSs allowed us to directly observe the distribution behavior *in vivo* and provide visual guidance for cancer treatment.

MRI images of the tumor sites were acquired at different time points after i.v. injection by ICON preclinical compact magnetic resonance imager. MnO_2_ could react with H_2_O_2_ to produce a large amount of paramagnetic Mn^2+^ in the TME, which would significantly enhance the T_1_ MRI signal. As expected, the T_1_-weighted imaging intensity was significantly higher than that of the surrounding tissues for the AAM-Ce6 HNSs treated group (Figure 7B), which was consistent with that of FL imaging. Moreover, as shown in Figure 7C, the MRI signal intensity of the tumor site gradually increased and reached a maximum at 12 hours, indicating the long-term imaging performance of AAM-Ce6 HNSs to tumors.

PAI images of the tumor sites were acquired at different time points postinjection by a preclinical photoacoustic scanner. As shown in Figure 7D, there was a significant PAI signal at the tumor site 12 hours after i.v. injection, implying that AAM-Ce6 HNSs had accumulated in the tumor sites. The PAI signal of the tumor sites gradually attenuated with time after 12 hours (Figure 7E) because the accumulated AAM-Ce6 HNSs could be gradually metabolized in mice. Combined with the abovementioned FL and MR imaging, AAM-Ce6 HNSs integrated three imaging modes (FL/MRI/PAI), which would be expected to provide long-term and accurate imaging guidance for the diagnosis and treatment of tumors.

### Effect of AAM-Ce6 HNSs on tumor oxygenation and PDT/PTT treatment *in vivo*

**Figure 8:**
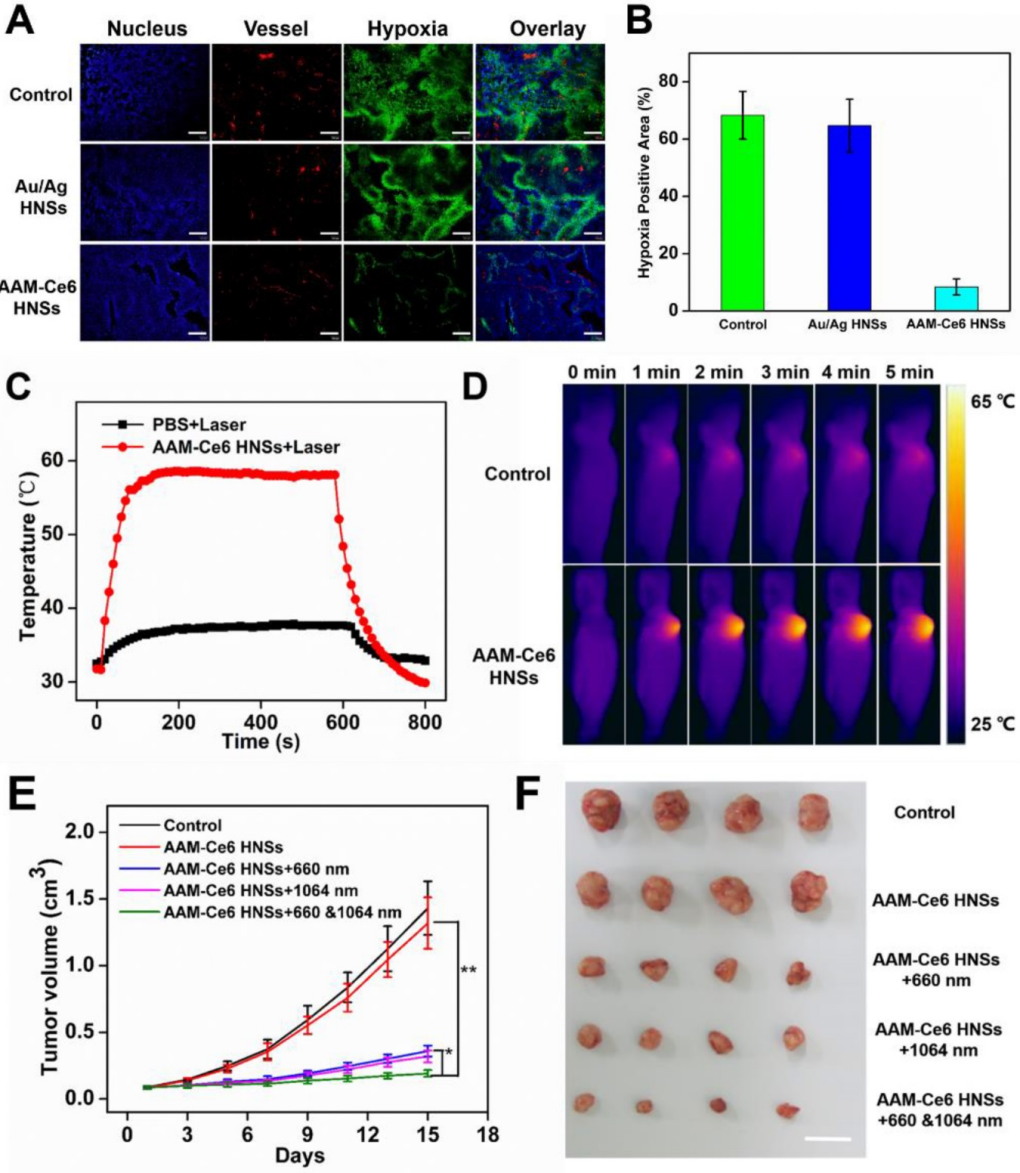
(A) Representative immunofluorescence images of tumor slices after hypoxia staining. The nuclei, blood vessels, and hypoxic areas were stained with DAPI (blue), anti-CD31 antibody (red), and anti-pimonidazole antibody (green), respectively. The scale bar is 200 μm. (B) The relative tumor hypoxia-positive areas of the different groups after treatment for 12 hours, determined from immunofluorescence images in (A). Temperature change curves (C) and photothermal images (D) of tumors over time after i.v. injection of PBS and AAM-Ce6 HNSs solutions radiated with a 1064 nm laser (1 W/cm^2^). Changes in tumor volume (E) and tumor images collected from mice with different treatment groups (F) 15 days after i.v. injection. The scale bar is 2 cm.

To further confirm that AAM-Ce6 HNSs have the ability to improve the tumor hypoxic environment, a hypoxia probe (pimonidazole) immunofluorescence assay was performed to examine tumor slices extracted after i.v. injection of different nanomaterials. It could be seen from the immunofluorescence images of tumor slices from tumor-bearing mice (Figure 8A), the nuclei, blood vessels, and hypoxic areas were stained with DAPI (blue), anti-CD31 antibody (red), and anti-pimonidazole antibody (green), respectively. Semi-quantitative analysis of hypoxia positive areas showed that the tumor treated with PBS remained highly hypoxic (positive area was 68.3%), the Au/Ag HNSs group (without of MnO_2_) could not effectively relieve the hypoxic state of tumor. In contrast, tumor-bearing mice treated with AAM-Ce6 HNSs (with MnO_2_) showed a significantly reduced area of hypoxia (positive area was 8.4% at 12 hours post injection) (Figure 8B).

HeLa tumor-bearing mice were randomly divided into five groups (four in each group) for PDT and PTT combined therapy experiments *in vivo*: (1) saline (as control), (2) AAM-Ce6 HNSs, (3) AAM-Ce6 HNSs +660 nm laser, (4) AAM-Ce6 HNSs +1064 nm laser, (5) AAM-Ce6 HNSs +660 nm and 1064 nm lasers. All mice were i.v. injected with either saline or AAM-Ce6 HNSs solution and continued to raise for 12 hours. The phototherapy groups were irradiated with a 660 nm laser (100 mW/cm^2^, 10 min) for PDT and a 1064 nm laser (1 W/cm^2^, 10 min) for PTT. The photothermal heating and cooling curves of mouse tumors were obtained (Figure 8C). Thermal images of HeLa tumor-bearing mice after i.v. injection with saline or AAM-Ce6 HNSs and irradiation with a 1064 nm laser (1 W/cm^2^) were recorded. As shown in Figure 8D, the temperature of tumor sites increased significantly in the AAM-Ce6 HNSs treatment group, but there was little change in the control group. The mice continued to be raised for 15 days, and the tumor weight, tumor volume and body weight of each mouse were recorded every two days. The mice were sacrificed on day 15, and the tumors were excised and weighed.

**Figure 9:**
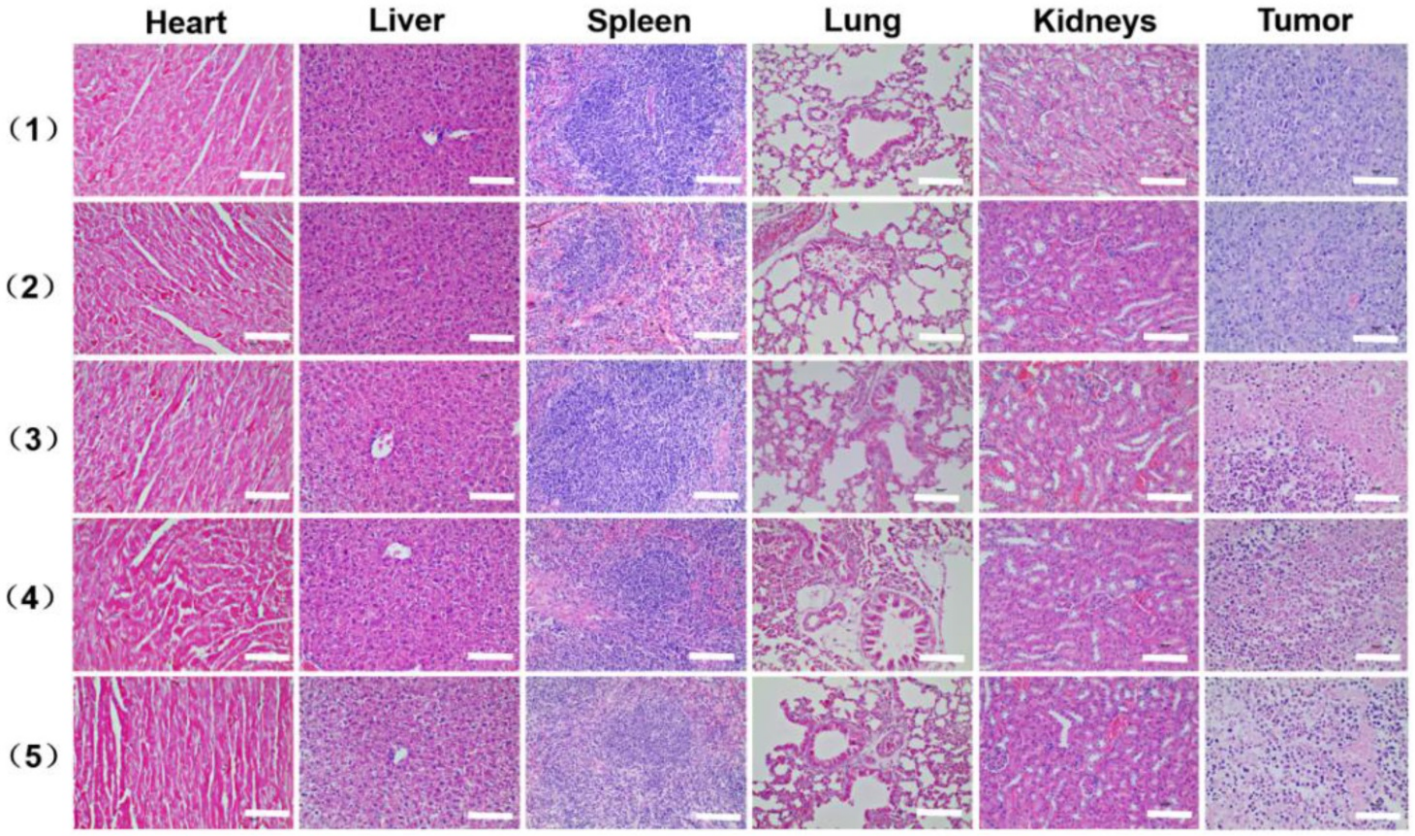
H&E stained images of the major organ and tumor tissue slices, including heart, liver, spleen, lung, kidney and tumor, collected from mice with different treatments after 15 days i.v. injection, (1) saline, (2) AAM-Ce6 HNSs, (3) AAM-Ce6 HNSs +660 nm laser, (4) AAM-Ce6 HNSs +1064 nm laser, (5) AAM-Ce6 HNSs +660 nm and 1064 nm lasers. The scale bar is 100 μm.

As shown in Figure 8E, the tumor volume increased gradually in the control and AAM-Ce6 HNSs groups without laser irradiation, indicating that there was no anticancer efficacy in saline and AAM-Ce6 HNSs treated without laser irradiation. Interestingly, both 660 nm laser and 1064 nm laser irradiation significantly suppressed tumor growth in the AAM-Ce6 HNSs +laser groups. The double lasers-treated group displayed the best inhibitory effect on tumor growth. Our results showed that the combination of PDT with PTT treatment reached 86.3 %, followed by PTT alone (75.7 %) and PDT alone (72.3 %). This proved that although PDT and PTT both had good therapeutic effects, they also had their own limitations. For PTT, an insufficient coverage area of light could lead to incomplete elimination of tumors, especially for tumor cells at the margin of tissues. For PDT, the photosensitizers easily aggregated and rapidly metabolized in the body, leading to inadequate treatment. Combining two therapies could promote and complement each other to achieve better therapeutic effects. Moreover, PDT could be improved by increasing the temperature of the photothermal effect. The systemic toxicity of AAM-Ce6 HNSs was assessed by the body weight of the mice. There was no obvious body weight drop in each treatment group compared to the control group, implying that no significant cytotoxicity was caused (Figure S11B). Isolated tumor weight and tumor images of different treatment groups after 15 days are shown in Figure S11A and Figure 8F, which further verified that AAM-Ce6 HNSs exhibited excellent photothermal and photodynamic effects for inhibiting tumor growth. Furthermore, hematoxylin and eosin (H&E) staining of major organ and tumor tissue slices were carried out after the mice were sacrificed. There were no noticeable damages in any of the major organs of the mice after 15 days of treatment. As expected, significant tumor cell damage could be observed in tumor sections of the AAM-Ce6 HNSs phototherapy-treated groups, indicating the important role of AAM-Ce6 HNSs in promoting tumor cell apoptosis (Figure 9).

## Conclusion

In summary, AAM-Ce6 HNSs have been successfully fabricated by a sacrifice template process and presented remarkable absorption in the NIR-II window. The AAM-Ce6 HNSs could be sensitive to TME with dual therapy functions of PTT and PDT. The HNSs showed outstanding photothermal conversion efficiency in the NIR-II window. AAM-Ce6 HNSs could catalyze endogenous H_2_O_2_ to produce O_2_ and enhance the therapeutic effect of PDT. The experimental results indicated that the combination of PDT and PTT could promote and complement each other and achieve a better overall therapeutic effect. Moreover, AAM-Ce6 HNSs had excellent FL/MR/PA trimodal imaging functions and provided good guidance for the treatment process. The AAM-Ce6 HNSs showed an efficient diagnostic and therapeutic effect for cancer treatment, which provides a new nanoplatform for antitumor research.

## Abbreviations

PTT: photothermal therapy
PDT: photodynamic therapy
KMnO_4_: permanganate
NIR: near infrared
MRI: magnetic resonance imaging
FL: fluorescence
PAI: photoacoustic imaging
MPE: maximum permissible exposure
ROS: reactive oxygen species
TME: tumor microenvironment
MnO_2_: manganese oxide
H_2_O_2_: hydrogen peroxide
AgNO_3_: Silver nitrate
C_6_H_8_O_6_: ascorbic acid
NaOH: sodium hydroxide
NaCl: sodium chloride
HAuCl_4_·3H_2_O: chloroauric acid trihydrate
KMnO_4_: potassium permanganate
PVP: polyvinyl pyrrolidon
DMSO: dimethyl sulfoxide
Ce6: chlorin e6
ABDA: 9, 10-anthracenediyl-bis (methylene) dimalonic acid
DCFH-DA: 2, 7-dichlorodihydrofluorescein diacetate
PEG-SH: thiol group polyethylene glycol
TEM: transmission electron microscopy
DLS: Dynamic light scattering
Ag NSs: silver nanospheres
AgCl: silver chloride
AAM HNSs: Au/Ag-MnO_2_ hollow nanospheres
DAPI: 4',6'-diamidino-2-phenylindole
MTT: methyl thiazolyl tetrazolium
i.v.: intravenously
IR: inhibition rate
H&E: hematoxylin and eosin
HAADF-STEM-EDX: high-angle annular dark-field scanning transmission electron microscopy energy-dispersive X-ray
SOSG: singlet oxygen sensor green probe

## References

[1] Ban Q, Bai T, Duan X, Kong J (2017). Noninvasive photothermal cancer therapy nanoplatforms via integrating nanomaterials and functional polymers. Biomater Sci.

[2] Zou L, Wang H, He B, Zeng L, Tan T, Cao H (2016). Current approaches of photothermal therapy in treating cancer metastasis with nanotherapeutics. Theranostics.

[3] Bashkatov AN, Genina EA, Kochubey VI, Tuchin VV (2005). Optical properties of human skin, subcutaneous and mucous tissues in the wavelength range from 400 to 2000 nm. J Phys D Appl Phys.

[4] Wang X, Ma Y, Sheng X, Wang Y, Xu H (2018). Ultrathin polypyrrole nanosheets via space-confined synthesis for efficient photothermal therapy in the second near-infrared window. Nano Lett.

[5] Tian Q, Jiang F, Zou R, Liu Q, Chen Z, Zhu M (2011). Hydrophilic Cu_9_S_5_ nanocrystals: A photothermal agent with a 25.7 % heat conversion efficiency for photothermal ablation of cancer cells *in vivo*. ACS Nano.

[6] Ji M, Xu M, Zhang W, Yang Z, Huang L, Liu J (2016). Structurally well-defined Au@Cu_2-x_S core-shell nanocrystals for improved cancer treatment based on enhanced photothermal efficiency. Adv Mater.

[7] Li A, Li X, Yu X, Li W, Zhao R, An X (2017). Synergistic thermoradiotherapy based on PEGylated Cu_3_BiS_3_ ternary semiconductor nanorods with strong absorption in the second near-infrared window. Biomaterials.

[8] Ding X, Liow CH, Zhang M, Huang R, Li C, Shen H (2014). Surface plasmon resonance enhanced light absorption and photothermal therapy in the second near-infrared window. J Am Chem Soc.

[9] Tsai MF, Hsu C, Yeh CS, Hsiao YJ, Su CH, Wang LF (2018). Tuning the distance of rattle-shaped IONP@shell-in-shell nanoparticles for magnetically-targeted photothermal therapy in the second near-infrared window. ACS Appl Mater Interfaces.

[10] Wang Z, Ju Y, Tong S, Zhang H, Lin J, Wang B (2018). Au_3_Cu tetrapod nanocrystals: highly efficient and metabolizable multimodality imaging-guided NIR-II photothermal agents. Nanoscale Horiz.

[11] Zhou J, Jiang Y, Hou S, Upputuri PK, Wu D, Li J (2018). Compact plasmonic blackbody for cancer theranosis in the near-infrared II window. ACS Nano.

[12] Cai K, Zhang W, Zhang J, Li H, Han H, Zhai T (2018). Design of gold hollow nanorods with controllable aspect ratio for multimodal imaging and combined chemo-photothermal therapy in the second near-infrared window. ACS Appl Mater Interfaces.

[13] Zhang W, Cai K, Li X, Zhang J, Ma Z, Foda MF (2019). Au hollow nanorods-chimeric peptide nanocarrier for NIR-II photothermal therapy and real-time apoptosis imaging for tumor heranostics. Theranostics.

[14] Jung HS, Verwilst P, Sharma A, Shin J, Sessler JL, Kim JS (2018). Organic molecule-based photothermal agents: an expanding photothermal therapy universe. Chem Soc Rev.

[15] Jiang Y, Li J, Zhen X, Xie C, Pu K (2018). Dual-peak absorbing semiconducting copolymer nanoparticles for first and second near-infrared window photothermal therapy: A comparative study. Adv Mater.

[16] Cao Y, Dou J-H, Zhao N-j, Zhang S, Zheng Y-Q, Zhang J-P (2016). Highly efficient NIR-II photothermal conversion based on an organic conjugated polymer. Chem Mater.

[17] Riley RS, Day ES (2017). Gold nanoparticle-mediated photothermal therapy: applications and opportunities for multimodal cancer treatment.

[18] Liu Y, Bhattarai P, Dai Z, Chen X (2019). Photothermal therapy and photoacoustic imaging via nanotheranostics in fighting cancer. Chem Soc Rev.

[19] Wang Q, Dai Y, Xu J, Cai J, Niu X, Zhang L (1901). All-in-one phototheranostics: single laser triggers NIR-II fluorescence/photoacoustic imaging guided photothermal/photodynamic/chemo combination therapy. Adv Funct Mater.

[20] Liu Y, Zhi X, Yang M, Zhang J, Lin L, Zhao X (2017). Tumor-triggered drug release from calcium carbonate-encapsulated gold nanostars for near-infrared photodynamic/photothermal combination antitumor therapy. Theranostics.

[21] Meng X, Liu Z, Cao Y, Dai W, Zhang K, Dong H (2017). Fabricating aptamer-conjugated PEGylated-MoS_2_/Cu_1.8_S theranostic nanoplatform for multiplexed imaging diagnosis and chemo-photothermal therapy of cancer. Adv Funct Mater.

[22] Huo D, Liu S, Zhang C, He J, Zhou Z, Zhang H (2017). Hypoxia-targeting, tumor microenvironment responsive nanocluster bomb for radical-enhanced radiotherapy. ACS Nano.

[23] Fan W, Yung B, Huang P, Chen X (2017). Nanotechnology for multimodal synergistic cancer therapy. Chem Rev.

[24] Agostinis P, Berg K, Cengel KA, Foster TH, Girotti AW, Gollnick SO (2011). Photodynamic therapy of cancer: an update. CA Cancer J Clin.

[25] Hirsch LR, Stafford RJ, Bankson JA, Sershen SR, Rivera B, Price RE (2003). Nanoshell-mediated near-infrared thermal therapy of tumors under magnetic resonance guidance. Proc Natl Acad Sci U S A.

[26] Zhang K, Zhang Y, Meng X, Lu H, Chang H, Dong H (2018). Light-triggered theranostic liposomes for tumor diagnosis and combined photodynamic and hypoxia-activated prodrug therapy. Biomaterials.

[27] Li X, Kwon N, Guo T, Liu Z, Yoon J (2018). Innovative strategies for hypoxic-tumor photodynamic therapy. Angew Chem Int Ed Engl.

[28] Cheng L, Yuan C, Shen S, Yi X, Gong H, Yang K (2015). Bottom-up synthesis of metal-ion-doped WS_2_ nanoflakes for cancer theranostics. ACS Nano.

[29] Cheng Y, Cheng H, Jiang C, Qiu X, Wang K, Huan W (2015). Perfluorocarbon nanoparticles enhance reactive oxygen levels and tumour growth inhibition in photodynamic therapy. Nat Commun.

[30] Wang J, Liu L, You Q, Song Y, Sun Q, Wang Y (2018). All-in-one theranostic nanoplatform based on hollow MoS_x_ for photothermally-maneuvered oxygen self-enriched photodynamic therapy. Theranostics.

[31] Song X, Xu J, Liang C, Chao Y, Jin Q, Wang C (2018). Self-supplied tumor oxygenation through separated liposomal delivery of H_2_O_2_ and catalase for enhanced radio-immunotherapy of cancer. Nano Lett.

[32] Wei J, Li J, Sun D, Li Q, Ma J, Chen X (2018). A novel theranostic nanoplatform based on Pd@Pt-PEG-Ce6 for enhanced photodynamic therapy by modulating tumor hypoxia microenvironment. Adv Funct Mater.

[33] Yang G, Xu L, Chao Y, Xu J, Sun X, Wu Y (2017). Hollow MnO_2_ as a tumor-microenvironment-responsive biodegradable nano-platform for combination therapy favoring antitumor immune responses. Nat Commun.

[34] Song M, Liu T, Shi C, Zhang X, Chen X (2016). Bioconjugated manganese dioxide nanoparticles enhance chemotherapy response by priming tumor-associated macrophages toward M1-like phenotype and attenuating tumor hypoxia. ACS Nano.

[35] Zhang X, Xi Z, Machuki JO, Luo J, Yang D, Li J (2019). Gold cube-in-cube based oxygen nanogenerator: A theranostic nanoplatform for modulating tumor microenvironment for precise chemo-phototherapy and multimodal imaging. ACS Nano.

[36] Lin T, Zhao X, Zhao S, Yu H, Cao W, Chen W (2018). O_2_-generating MnO_2_ nanoparticles for enhanced photodynamic therapy of bladder cancer by ameliorating hypoxia. Theranostics.

[37] Shin J, Anisur RM, Ko MK, Im GH, Lee JH, Lee IS (2009). Hollow manganese oxide nanoparticles as multifunctional agents for magnetic resonance imaging and drug delivery. Angew Chem Int Ed Engl.

[38] Hu D, Chen L, Qu Y, Peng J, Chu B, Shi K (2018). Oxygen-generating hybrid polymeric nanoparticles with encapsulated doxorubicin and chlorin e6 for trimodal imaging-guided combined chemo-photodynamic therapy. Theranostics.

[39] Liang R, Liu L, He H, Chen Z, Han Z, Luo Z (2018). Oxygen-boosted immunogenic photodynamic therapy with gold nanocages@manganese dioxide to inhibit tumor growth and metastases. Biomaterials.

[40] Yi X, Chen L, Zhong X, Gao R, Qian Y, Wu F (2016). Core-shell Au@MnO_2_ nanoparticles for enhanced radiotherapy via improving the tumor oxygenation. Nano Res.

[41] Yao Y, Zhao D, Li N, Shen F, Machuki JO, Yang D (2019). Multifunctional Fe_3_O_4_@polydopamine@DNA-fueled molecular machine for magnetically targeted intracellular Zn^2+^ imaging and fluorescence/MRI guided photodynamic-photothermal therapy. Anal Chem.

[42] Zhu J, Lu Y, Li Y, Jiang J, Cheng L, Liu Z (2014). Synthesis of Au-Fe_3_O_4_ heterostructured nanoparticles for *in vivo* computed tomography and magnetic resonance dual model imaging. Nanoscale.

[43] Wu J, Li N, Yao Y, Tang D, Yang D, Ong'achwa Machuki J (2018). DNA-stabilized silver nanoclusters for label-free fluorescence imaging of cell surface glycans and fluorescence guided photothermal therapy. Anal Chem.

[44] Zhang K, Yang Z, Meng X, Cao Y, Zhang Y, Dai W (2018). Peroxidase-like Fe_3_O_4_ nanocomposite for activatable reactive oxygen species generation and cancer theranostics. Mater Chem Front.

[45] Cao Y, Meng X, Wang D, Zhang K, Dai W, Dong H (2018). Intelligent MnO_2_/Cu_2- x_S for multimode imaging diagnostic and advanced single-laser irradiated photothermal/photodynamic therapy. ACS Appl Mater Interfaces.

[46] Chen B, Jiao X, Chen D (2010). Size-controlled and size-designed synthesis of nano/submicrometer Ag particles. Cryst Growt Des.

[47] Lu X, Au L, McLellan J, Li ZY, Marquez M, Xia Y (2007). Fabrication of cubic nanocages and nanoframes by dealloying Au/Ag alloy nanoboxes with an aqueous etchant based on Fe(NO_3_)_3_ or NH_4_OH. Nano Lett.

[48] Liao J, Wei X, Ran B, Peng J, Qu Y, Qian Z (2017). Polymer hybrid magnetic nanocapsules encapsulating IR820 and PTX for external magnetic field-guided tumor targeting and multifunctional theranostics. Nanoscale.

[49] Dong Z, Feng L, Hao Y, Chen M, Gao M, Chao Y (2018). Synthesis of hollow biomineralized CaCO_3_-polydopamine nanoparticles for multimodal imaging-guided cancer photodynamic therapy with reduced skin photosensitivity. J Am Chem Soc.

[50] Dai Y, Su J, Wu K, Ma W, Wang B, Li M (2019). Multifunctional thermosensitive liposomes based on natural phase-change material: Near-infrared light-triggered drug release and multimodal imaging-guided cancer combination therapy. ACS Appl Mater Interfaces.

[51] Ling Y, Zhang D, Cui X, Wei M, Zhang T, Wang J (2019). Direct monitoring cell membrane vesiculation with 2D AuNP@MnO_2_ nanosheet supraparticles at single-particle level. Angew Chem Int Ed Engl.

[52] Li S, Zhang L, Chen X, Wang T, Zhao Y, Li L (2018). Selective growth synthesis of ternary janus nanoparticles for imaging-guided synergistic chemo- and photothermal therapy in the second NIR window. ACS Appl Mater Interfaces.

[53] Bai Y-H, Du Y, Xu J-J, Chen H-Y (2007). Choline biosensors based on a bi-electrocatalytic property of MnO_2_ nanoparticles modified electrodes to H_2_O_2_. Electrochem commun.

[54] Szatrowski TP, Nathan CF (1991). Production of large amounts of hydrogen peroxide by human tumor cells. Cancer Res.

[55] Gao DY, Ji X, Wang JL, Wang YT, Li DL, Liu YB (2018). Engineering a protein-based nanoplatform as an antibacterial agent for light activated dual-modal photothermal and photodynamic therapy of infection in both the NIR I and II windows. J Mater Chem B.

[56] Yang G, Sun X, Liu J, Feng L, Liu Z (2016). Light-responsive, singlet-oxygen-triggered on-demand drug release from photosensitizer-doped mesoporous silica nanorods for cancer combination therapy. Adv Funct Mater.

[57] Mikawa M, Kato H, Okumura M, Narazaki M, Kanazawa Y, Miwa N (2001). Paramagnetic water-soluble metallofullerenes having the highest relaxivity for MRI contrast agents. Bioconjug Chem.

[58] Wang LV, Hu S (2012). Photoacoustic tomography: *in vivo* imaging from organelles to organs. Science.

