# Supplementary material for: Endogenous oxygen generating multifunctional theranostic nanoplatform for enhanced photodynamic-photothermal therapy and multimodal imaging
Source: Theranostics. 2019 Oct 15;9(25):7697–713. doi: 10.7150/thno.38565 (PMC6831477; doi:10.7150/thno.38565)
Supplement: Supplementary file 1 — Supplementary figures and tables. [file thnov09p7697s1.pdf]

Supplementary file

Endogenous oxygen generating multifunctional theranostic nanoplatform for enhanced photodynamic-photothermal therapy and multimodal imaging

Kun Wu[1], Honghai Zhao[1], Zhiquan Sun[1], Bing Wang[1], Xueying Tang[1] Yeneng Dai[1], Meixing Li[1], Qingming Shen[1,*], Hui Zhang[1, 2,*], Quli Fan[1,*], Wei Huang[1, 3]

Affiliations:

1 Key Laboratory for Organic Electronics and Information Displays & Jiangsu Key Laboratory for Biosensors, Institute of Advanced Materials (IAM), Jiangsu National Synergetic Innovation Center for Advanced Materials (SICAM), Nanjing University of Posts & Telecommunications, Nanjing 210023, China.

2 Jiangsu Key Laboratory of New Power Batteries, College of Chemistry and Materials Science, Nanjing Normal University, Nanjing 210097, China

3 Shaanxi Institute of Flexible Electronics (SIFE), Northwestern Polytechnical University, Xi'an 710072, China

*** Corresponding authors:**

Qingming Shen, Ph.D.

Email: iamqmshen@njupt.edu.cn

Hui Zhang, Ph.D.

Email: zhangh@njnu.edu.cn

Quli Fan, Ph.D.

Email: iamqlfan@njupt.edu.cn

Measurement of photothermal conversion efficiency

The AAM-Ce6 HNSs solution with a concentration of 40 μg/mL was used to calculate the photothermal conversion efficiency. Briefly, the sample solution was irradiated at 808 nm or 1064 nm for 10 minutes, then the laser was turned off, and the solution allowed to cool naturally for 10 minutes. The photothermal conversion efficiency was calculated using formula (a):

$$\eta = \frac{hS(T_{max} - T_{surr}) - Q_{dis}}{I(1 - 10^{-A_\lambda})} \qquad \text{(a)}$$

where *h* respresents the heat transfer coefficient, *S* is the surface area of the container, T_{Surr} is the temperature of the surrounding environment, T_{Max} is the temperature at which the solution reaches a steady state under continuous irradiation, *I* is the power density of the laser (1 W/cm^2), *A* is the absorbance of AAM-Ce6 HNSs at 808 nm and 1064 nm. Q_{Dis} indicates the heat loss of the light absorbed by the quartz sample cell. *hS* is caculated by the formula (b):

$$hS = \frac{mC_p}{\tau_s} \qquad \text{(b)}$$

where *m*, C_p, are the mass, heat capacity of water, and τ_s is sample system time constant, which can be obtained according to formula (c):

$$t = -\tau_s In\theta \qquad \text{(c)}$$

where *t* is the time value corresponding to each point in the cooling process starting from highest temperature, θ is caculated by the formula (d):

$$\theta = \frac{T - T_{surr}}{T_{max} - T_{surr}} \qquad \text{(d)}$$

T represents the temperature corresponding to each time point during the cooling process.

Measurement of blocking effects (%)

In order to explore the tissue penetration depth in the NIR-II windows and NIR-I windows. The temperature changes of AAM-Ce6 HNSs solution covered by chicken breasts with different thicknesses were recorded under 1064 nm and 808 nm laser irradiation. The blocking effects was calculated using formula (e):

$$\text{blocking effects (\%)} = \frac{T_{before} - T_{after}}{T_{before}} \qquad \text{(e)}$$

T_{before} and T_{after} are the temperature before and after covering different thicknesses of chicken breast, respectively.

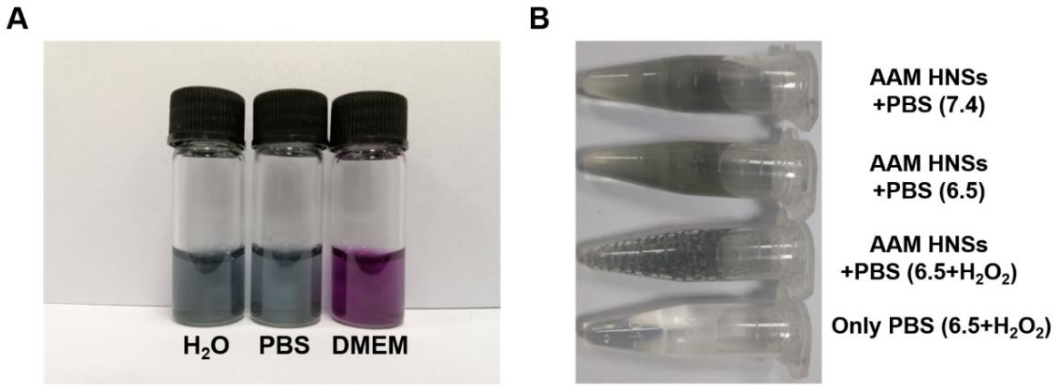

Figure S1 (A) Picture of AAM-Ce6 HNSs in various aqueous media: water, PBS and DMEM cell medium containing 10 % fetal bovine serum (FBS). (B) Picture of the oxygen generation in different solutions, with H_2O_2 concentration 10 mM.

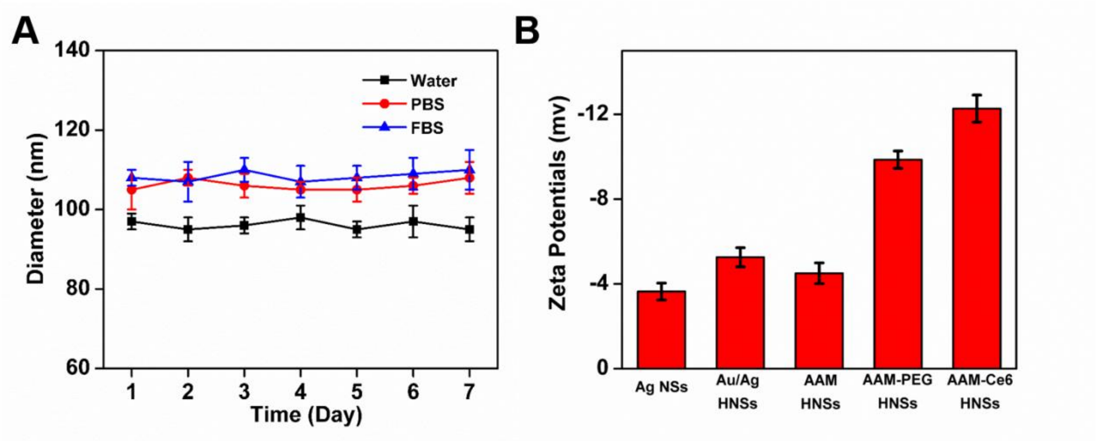

Figure S2 (A) The hydrodynamic diameters fluctuation of the AAM-Ce6 HNSs stored in water, PBS and FBS for different time periods. (B) The changes of zeta potentials for nanoparticles obtained at different steps of fabrication.

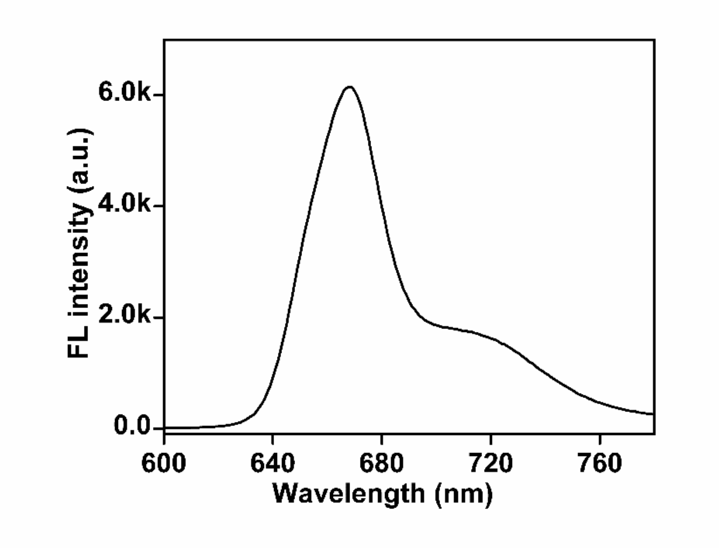

Figure S3 The fluorescence spectrum of AAM-Ce6 HNSs when excited at the wavelength of 420 nm.

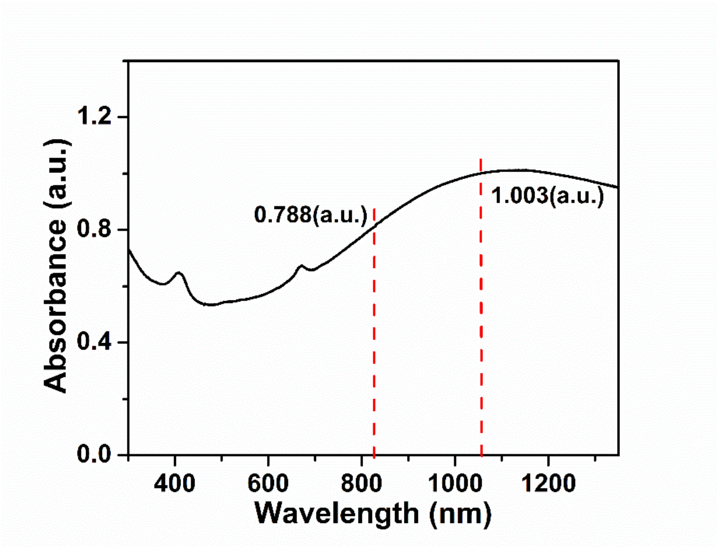

Figure S4 UV-vis-NIR absorption spectra of AAM-Ce6 HNSs (40 μg/mL). The red dashed lines are the absorption intensity at 808 and 1064, respectively.

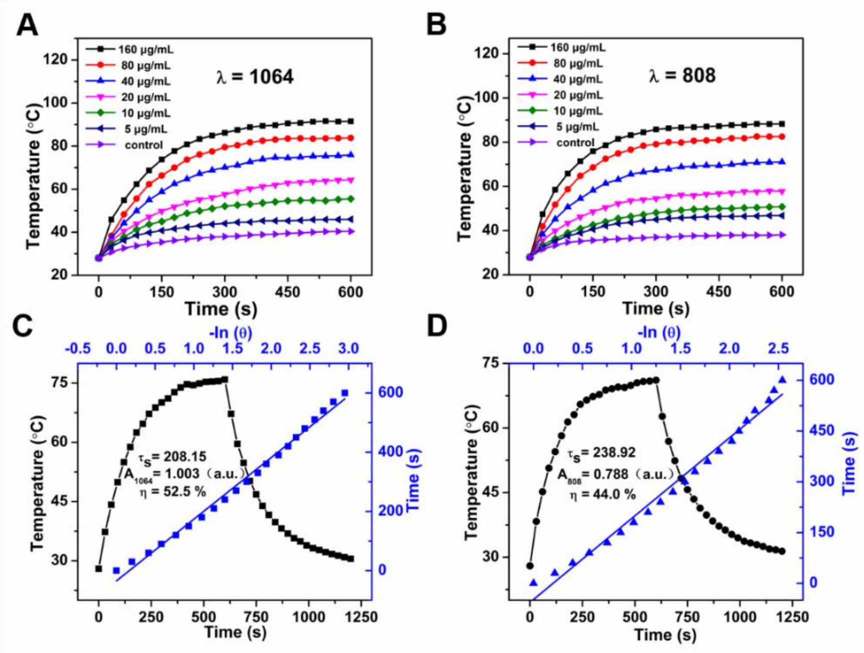

Figure S5 Photothermal effect of AAM-Ce6 HNSs irradiated under a 1064 nm or 808 nm laser with a power density of 1 W/cm^2. Temperature changes with different concentrations of AAM-Ce6 HNSs solution under irradiation of 1064 nm laser (A) and 808 nm laser (B) for 10 minutes. The heating and cooling curve (black) and the solution cooling time and -In (θ) linear relationship (blue) of AAM-Ce6 HNSs solution (40 μg/mL) under the radiation of 1064 nm laser (C) and 808 nm laser (D).

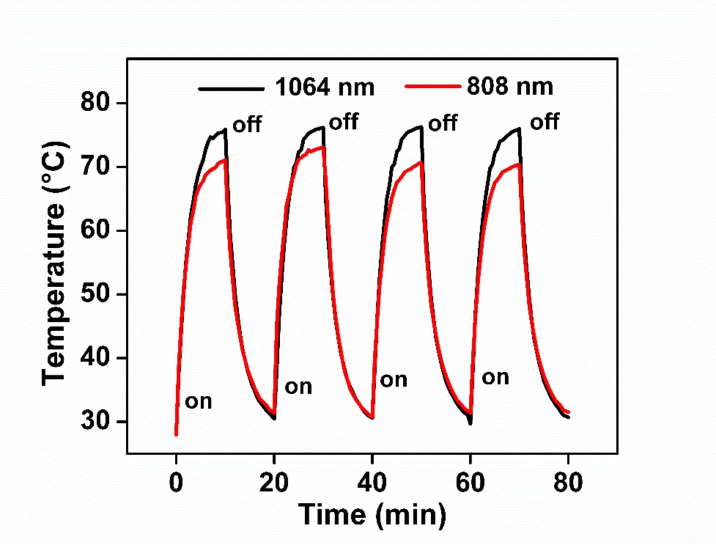

Figure S6 Cycle heating/cooling profiles of aqueous solutions containing AAM-Ce6 HNSs (40 μg/mL) at the irradiation of 1064 nm and 808 nm laser (1 W/cm^2).

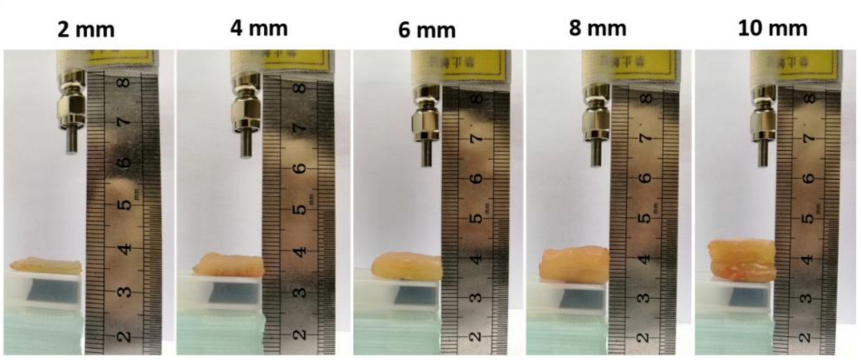

Figure S7 Experimental scene of samples covered by chicken breasts of different thicknesses.

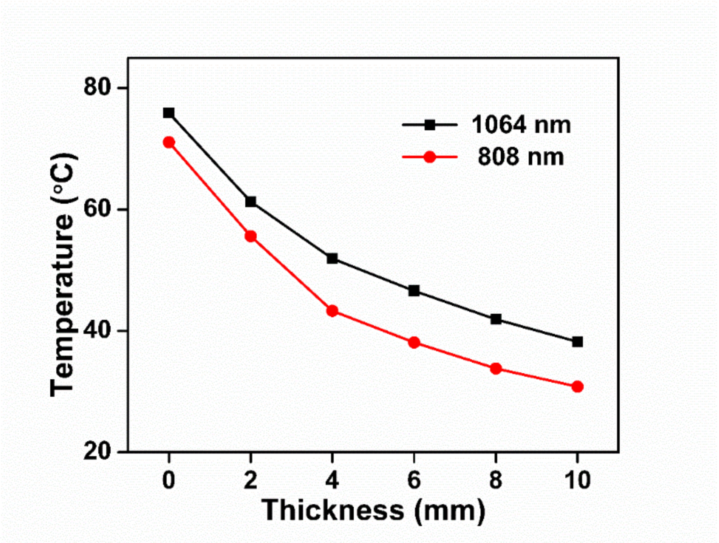

Figure S8 The temperature changes of AAM-Ce6 HNSs (40 μg/mL) covered with different thicknesses of chicken breast upon exposure to 1064 nm or 808 nm laser for 10 minutes.

Table S1 Different temperature blocking effects (%) covered with chicken breast of different thickness for the same radiation time.

Laser	0 mm	2 mm	4 mm	6 mm	8 mm	10 mm
**808 nm**	0	21.8	39.1	46.4	52.5	56.7
**1064 nm**	0	14.6	31.6	38.6	44.8	49.7

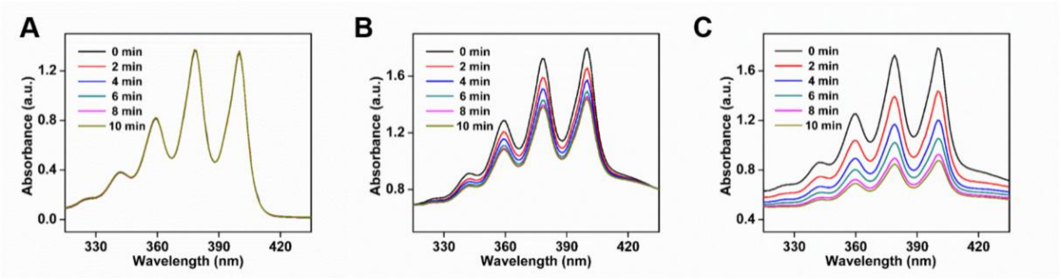

Figure S9 UV-vis absorption spectra of ABDA in different samples under irradiation with a 660 nm laser (100 mW/cm^2 10 min): (A) PBS 5.5; (B) AAM-Ce6 HNSs in PBS 7.4; (C) AAM-Ce6 HNSs in PBS 5.5. The final concentration of H_2O_2 was 100 μM.

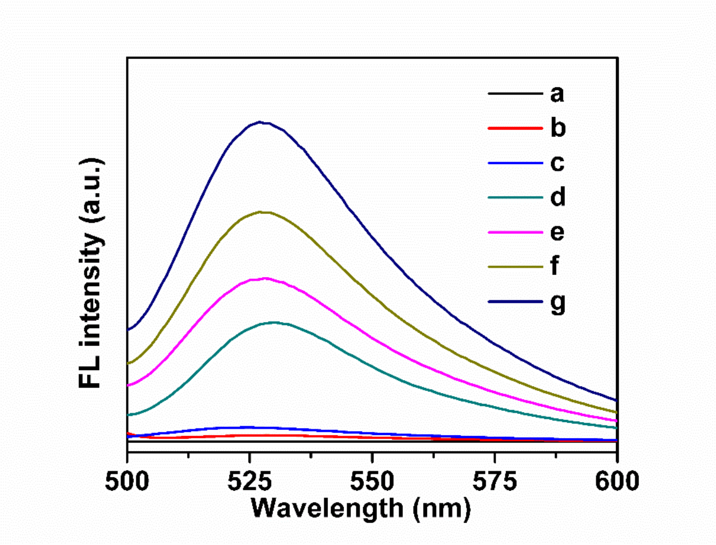

Figure S10 Singlet Oxygen Sensor Green (SOSG) fluorescence spectra of different groups. (a) PBS +660 nm, (b) SOSG +660 nm, (c) AAM-Ce6 HNSs +SOSG +1064 nm, (d) AAM-Ce6 HNSs +SOSG +660 nm, (e) AAM-Ce6 HNSs +SOSG +1064 nm and 660 nm, (f) AAM-Ce6 HNSs +SOSG +660 nm (pH 5.5, H_2O_2), (g) AAM-Ce6 HNSs +SOSG +1064 nm and 660 nm (pH 5.5, H_2O_2). The final concentration of H_2O_2 was 100 μM.

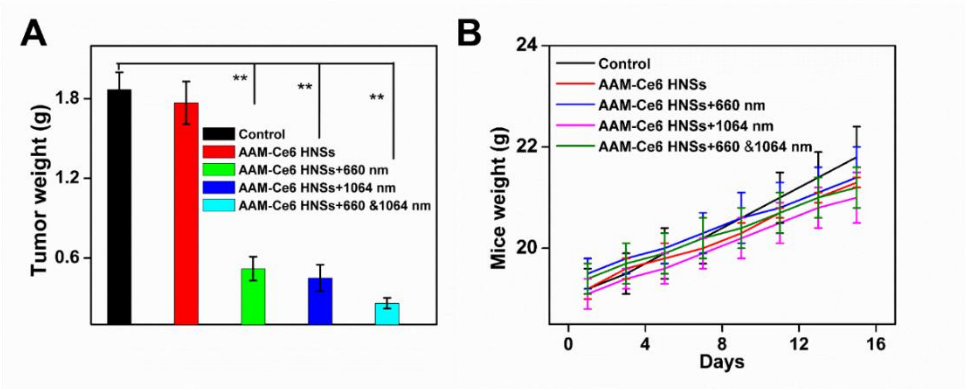

Figure S11 Changes in tumor weight (A) and mice weight (B) of different treatment groups.
